# Von Willebrand Factor Mediates Pneumococcal Aggregation and Adhesion in Blood Flow

**DOI:** 10.3389/fmicb.2019.00511

**Published:** 2019-03-26

**Authors:** Hilger Jagau, Ina-Kristin Behrens, Karen Lahme, Georgina Lorz, Reinhard W. Köster, Reinhard Schneppenheim, Tobias Obser, Maria A. Brehm, Gesa König, Thomas P. Kohler, Manfred Rohde, Ronald Frank, Werner Tegge, Marcus Fulde, Sven Hammerschmidt, Michael Steinert, Simone Bergmann

**Affiliations:** ^1^Institute of Microbiology, Technische Universität Braunschweig, Braunschweig, Germany; ^2^Zoological Institute, Technische Universität Braunschweig, Braunschweig, Germany; ^3^Department of Pediatric Hematology and Oncology, University Medical Center Hamburg-Eppendorf (UKE Hamburg), Hamburg, Germany; ^4^Department of Molecular Genetics and Infection Biology, Interfaculty Institute for Genetics and Functional Genomics, Universität Greifswald, Greifswald, Germany; ^5^Helmholtz Centre for Infection Research, Central Facility for Microscopy, Braunschweig, Germany; ^6^Department of Chemical Biology, Helmholtz Centre for Infection Research, Braunschweig, Germany; ^7^Centre for Infection Medicine, Institute of Microbiology and Epizootics, Freie Universität Berlin, Berlin, Germany; ^8^Department of Molecular Infection Biology, Helmholtz Centre for Infection Research, Braunschweig, Germany

**Keywords:** endothelium, von Willebrand factor, enolase, *Streptococcus pneumoniae*, zebrafish

## Abstract

*Streptococcus pneumoniae* is a major cause of community acquired pneumonia and septicaemia in humans. These diseases are frequently associated with thromboembolic cardiovascular complications. Pneumococci induce the exocytosis of endothelial Weibel-Palade Bodies and thereby actively stimulate the release of von Willebrand factor (VWF), which is an essential glycoprotein of the vascular hemostasis. Both, the pneumococcus induced pulmonary inflammation and the thromboembolytic complications are characterized by a dysbalanced hemostasis including a marked increase in VWF plasma concentrations. Here, we describe for the first time VWF as a novel interaction partner of capsulated and non-encapsulated pneumococci. Moreover, cell culture infection analyses with primary endothelial cells characterized VWF as bridging molecule that mediates bacterial adherence to endothelial cells in a heparin-sensitive manner. Due to the mechanoresponsive changes of the VWF protein conformation and multimerization status, which occur in the blood stream, we used a microfluidic pump system to generate shear flow-induced multimeric VWF strings on endothelial cell surfaces and analyzed attachment of RFP-expressing pneumococci in flow. By applying immunofluorescence visualization and additional electron microscopy, we detected a frequent and enduring bacterial attachment to the VWF strings. Bacterial attachment to the endothelium was confirmed *in vivo* using a zebrafish infection model, which is described in many reports and acknowledged as suitable model to study hemostasis mechanisms and protein interactions of coagulation factors. Notably, we visualized the recruitment of zebrafish-derived VWF to the surface of pneumococci circulating in the blood stream and detected a VWF-dependent formation of bacterial aggregates within the vasculature of infected zebrafish larvae. Furthermore, we identified the surface-exposed bacterial enolase as pneumococcal VWF binding protein, which interacts with the VWF domain A1 and determined the binding kinetics by surface plasmon resonance. Subsequent epitope mapping using an enolase peptide array indicates that the peptide ^181^YGAEIFHALKKILKS^195^ might serve as a possible core sequence of the VWF interaction site. In conclusion, we describe a VWF-mediated mechanism for pneumococcal anchoring within the bloodstream via surface-displayed enolase, which promotes intravascular bacterial aggregation.

## Introduction

In the course of systemic infection, *Streptococcus pneumoniae* enters various tissue sites including bronchial airways, lung alveoli and the blood stream (Bergmann and Hammerschmidt, 2006; Weiser et al., 2018). Thus, in young children, immunocompromised and elderly humans, pneumococci cause local infections but also severe systemic diseases, such as community acquired pneumonia (CAP), meningitis and septicemia (Cartwright, 2002). Cardiovascular diseases are regularly reported complications of pneumococcal pneumonia and septicemia. During the last decade, an increasing amount of medical studies from the US and different regions in Europe reported consistently that up to one third of patients suffer from major adverse cardiac effects and vascular impairments within months and even years after recovering from severe pneumococcal infections (Musher et al., 2007; van Schie et al., 2011; Corrales-Medina et al., 2015; Rae et al., 2016). Moreover, pneumococci have been identified as causative agent for vascular inflammation of the endothelial vessel wall including the aorta (Sakai et al., 2000). In this respect, several formerly published reports demonstrated adherence of *S. pneumoniae* to human endothelial cells (EC's), which is promoted by extracellular matrix and plasma proteins, such as vitronectin (Bergmann et al., 2009) and plasminogen (Bergmann et al., 2013). We previously reported that the pneumococcus cell adherence to the vascular lung endothelium immediately triggers the induction of endothelial Weibel-Palade Body (WPB) exocytosis (Luttge et al., 2012). Vascular homeostasis is regulated by WPB's (Weibel and Palade, 1964; Rondaij et al., 2004), which constitute specific defense vesicles of human ECs and are mainly composed of von Willebrand Factor (VWF), pro-angiogenic proteins, cytokines and vasoactive substances (Ruggeri, 2001; Rondaij et al., 2006).

VWF circulates as a globular glycoprotein in the bloodstream (Ruggeri, 2001) and also acts as plasma-carrier protein for coagulation factor VIII (Ruggeri, 2001; Rondaij et al., 2006). In addition to the circulating globular form of VWF, high molecular weight (HMW) multimers can form strings of up to several hundred micrometer in length on the surface of stimulated EC's or when attached to collagen exposed by vascular injury in response to hydrodynamic forces generated by the bloodstream. In this extended conformation, VWF plays a key role in hemostasis by mediating adhesion of platelets to damaged or inflamed vascular subendothelium (Tischer et al., 2017). VWF is of high importance for a balanced hemostasis, since quantitative or functional VWF deficiency causes von Willebrand disease, a common inherited bleeding disorder (Ruggeri, 2001). Likewise, VWF concentration in the blood typically rises during the course of acute coronary syndrome, and is an independent predictor of adverse clinical outcome in these patients (Spiel et al., 2008). Various lines of evidence indicate that VWF is not only a marker but also actually an important effector in the pathogenesis of myocardial infarction (Spiel et al., 2008). As a multi-domain mosaic protein, VWF possesses several functionally important domains, such as the central domains A1 and A3. These domains are pivotal for platelet recruitment and are involved in anchoring of VWF to collagen (Springer, 2014).

To understand the consequences of pneumococcus-induced secretion of VWF for bacterial interaction with the endothelium and possible implications in development of cardiovascular disorders, we analyzed in detail the interaction of pneumococci with plasma-derived VWF and also with VWF strings on the surface of endothelial cells. We identified and characterized the enolase as the bacterial VWF binding protein and determined the role of VWF as an adhesion cofactor promoting bacterial attachment to the vascular endothelium. We monitored VWF-pneumococcus colocalization and VWF-mediated bacterial aggregation *in vivo* in zebrafish larvae. The zebrafish infection model is highly suitable for our study, since bacterial infection of zebrafish is commonly approved as an adequate *in vivo* model to study the pathophysiology of various streptococcal infections including pneumococcal diseases (Miller and Neely, 2004; Saralahti et al., 2014; Saralahti and Rämet, 2015; Jim et al., 2016). Moreover, it served as object of preference for hemostasis and thrombosis studies due to high functional similarities of coagulation cascades and additional high sequence based similarities between zebrafish and human factors including VWF (Weyand and Shavit, 2014).

Overall, our results underpin the function of VWF as a coadhesin mediating attachment of pneumococci to the endothelium in the blood flow. Furthermore, our *in vivo* data visualize a VWF-mediated intravascular bacterial aggregation, which suggests a pivotal role of VWF in reported long term effects of pneumococcus pneumoniae and systemic infections.

## Materials and Methods

### Bacterial Strains and Cultivation

VWF binding analyses were performed with seven different pneumococcal strains including the laboratory strains: R6, R800, TIGER4 (serotype 4), D39 (serotype 2), and serotype 35A, and the clinical isolates of serotypes 23F and 12F. The strains differ in the amount of polysaccharide capsule as indicated in results. Red fluorescent protein (RFP)-expressing serotype 35A pneumococci were generated essentially as described in Kjos et al. (2015). This serotype was also used in all cell culture infection assays, microfluidic analyses and *in vivo* zebrafish infections. For cell culture infection and binding analyses, pneumococci were cultured on Columbia blood agar (Becton Dickinson) to mid-log phase (OD_600_ of 0.35) at 37°C and 5.0% CO_2_ or in Todd-Hewitt broth (Becton Dickinson) supplemented with 1.0% yeast extract (THY).

### Proteins and Enzymes

Human VWF was purchased from Merck Chemicals; histamine from Sigma Aldrich, Germany. The recombinant VWF domains (A1, A2, and A3) were produced in HEK293 cells (Posch et al., 2017). Pneumococcus-specific polyclonal antiserum was generated in rabbit against heat inactivated serotype 35A and 2 bacteria (Pineda, Germany). IgG was purified using protein A/G sepharose. Mouse anti-human VWF antibody (polyclonal IgG) and FITC-conjugated VWF-specific antibody were purchased from Abcam (ab8822), peroxidase-conjugated mouse anti-VWF antibody from Hämochrom Diagnostika; Alexa Fluor 488-conjugated goat anti-rabbit and goat anti-mouse IgG, Alexa Fluor 568-conjugated goat anti-rabbit and goat anti-mouse IgG were from Thermo Fisher Scientific (formerly Invitrogen), paraformaldehyde (PFA) was purchased from EM Science and mounting medium from Dako.

### Bacterial Binding to Radiolabeled VWF

Human VWF was radiolabeled with ^125^iodine-isotope by a standard chloramine T method and binding experiments with [^125^I]-VWF were performed as described previously (Bergmann et al., 2001). Briefly, 10^9^ bacteria grown to mid-log phase were incubated with 20 nCi of radiolabeled VWF for 30 min at RT. Pneumococci were sedimented by centrifugation and after washing twice, pellet-bound radioactivity representing bound VWF was measured in a gamma counter (Packard). VWF binding was expressed as a percentage of total added radioactivity. The labelling efficiency was calculated using fetal calf serum and used to define the level of unspecific binding. The shown binding data were normalized by subtraction of this level.

### *In vitro* Cell Culture Infection

Cell culture infection analyses were performed according to a previously described standard procedure using serotype 35A pneumococci grown to mid log phase (Bergmann et al., 2001). This strain produces all essential traits of virulence and a minor amount of capsule and is a commonly used model strain for cell culture infection analyses. A detailed description of cell cultivation and infection methodology is included in Supplementary Material. In brief, after incubation of HUVEC with different concentrations of VWF, the cells were incubated with pneumococci for 3 h. Differential immunofluorescence labeling enabled quantification of attached and internalized bacteria as reported by Lüttge and colleges (Luttge et al., 2012). For inhibition studies, either HUVEC or bacteria were incubated with 30 IU heparin and with 3 μg/ml VWF. As control, cells were incubated with bacteria alone or with VWF-coated bacteria without inhibitor substance. For control of inoculum, bacteria were plated on THY- solid agar medium. Statistical significances were analyzed by the ANOVA one –factorial test in case of the infection analyses with different VWF concentrations and the two-factorial variance analysis in case of the heparin-inhibition analysis followed by a *post-hoc* two-tailed unpaired sample test for detailed statistical comparison. *P*-values of <0.05 were considered to be statistically significant.

### Flow Cultivation and Infection

Flow cultivation was performed using the microfluidic system of ibidi® (Munich, Germany) and RFP-expressing serotype 35A pneumococci grown to mid log phase. In brief, primary HUVEC were seeded on gelatin-coated 0.4 mm μ-Slides (μ-Slide I^0.4^ Luer, ibidi®) and cultured in continuous flow of 5 dyne/cm^2^ for 30 min followed by 10 dyne/cm^2^ for 48 h. Prior to incubation for up to 3 h with 10^8^ RFP-expressing bacteria per ml, VWF secretion was stimulated with 1.0 mM histamine. For more detailed experimental procedure refer to Supplementary Methods. Generation of VWF strings and bacterial adherence was microscopically documented for up to 3 h using a FITC-conjugated, polyclonal anti-VWF antibody. All experiments were performed in three independent assays, each in triplicate and the data were expressed as mean standard deviation.

### Microscopic Illustrations

All microscopic images were taken with a Xenon fluorescence device (30% power) of a SP8 confocal laser scanning microscope (Leica) combined with the LasX-Software using a HC PL APO CS2 63 x/1.40 oil objective and a resolution of 1,392 × 1,040 pixel. Brightness and contrast were adapted using Adobe Photoshop CS5 (version 12.0.). For microscopic visualization of zebrafish infection, a HC PL APO CS2 20 × /0.75 IMM objective was used at a resolution of 1,392 × 1,040 pixel. Blood circulation in zebrafish was monitored as video using 4 frames/min with a resolution of 1,392 × 1,040 pixels.

### Electron Microscopic Visualization

**Figure 1:**
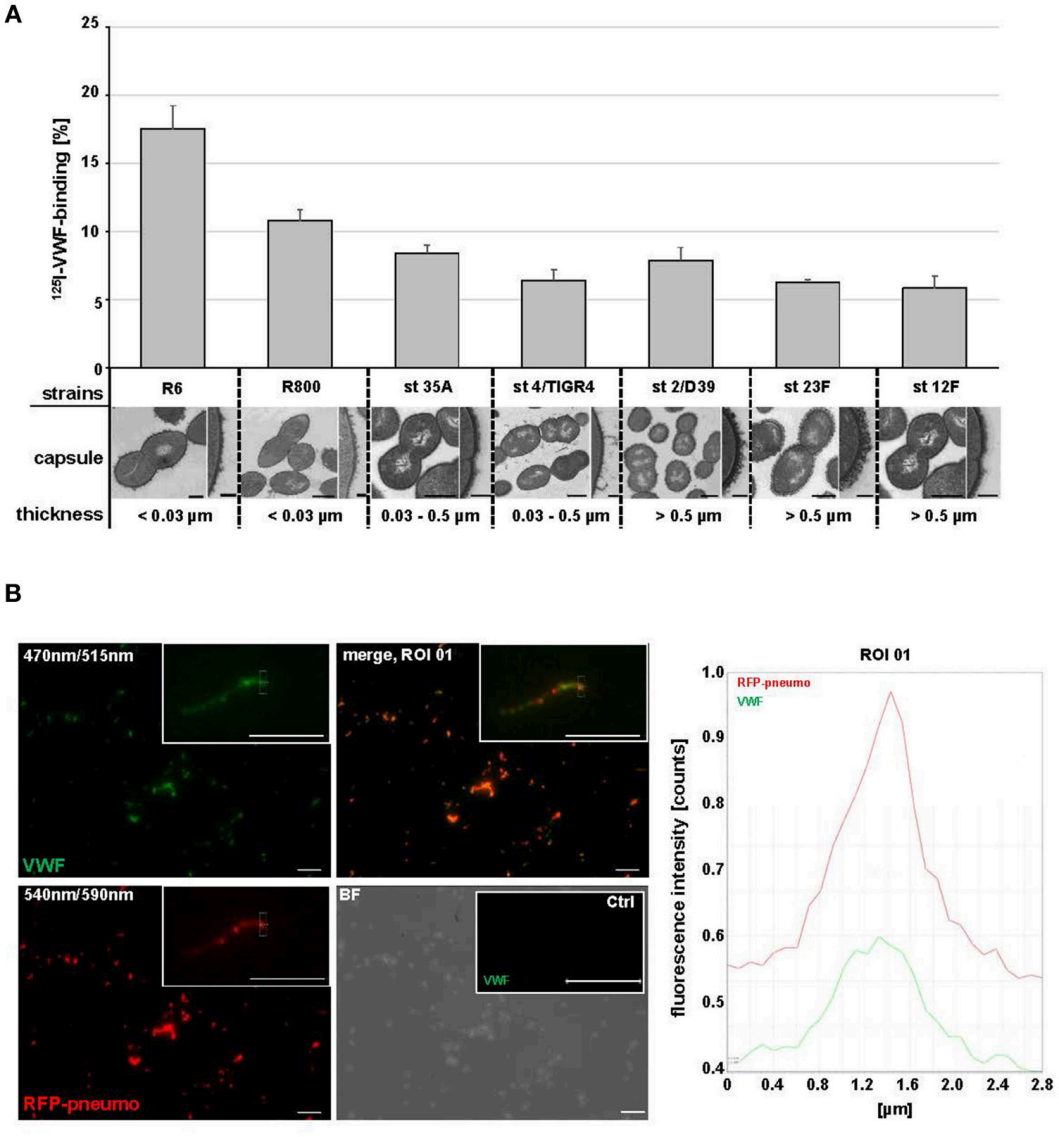
*Streptococcus pneumoniae* binds human plasma VWF. **(A)** Binding analyses with radioactive labeled VWF and seven *S. pneumoniae* strains: R6, R800 and the serotypes (st) 35A, 4 (TIGER4), 2 (D39), 23F and 12F. VWF binding was expressed as percentage of total radioactivity added. Amount of capsular polysaccharides (CPS) was categorized according to the CPS-diameter determined by electron microscopic visualization: low encapsulation <0.03μm, middle encapsulation in a range of 0.03–0.5μm and high encapsulation >0.5μm. Scale bars represent 0.5 μm in the overview figures and 0.1 μm in magnified sections. **(B)** Immunofluorescence visualization of VWF binding to RFP-expressing pneumococci with FITC-conjugated VWF-specific antibodies after embedding of VWF-incubated pneumococci in agarose. RFP-expressing bacteria appear in red at 540/590 nm and VWF-detection is visualized in green at 470/515 nm. VWF coated bacteria appear in yellow in the merged channel. The insets visualize a representative fluorescence signal at 3-fold magnification. Antibody controls revealed no unspecific VWF-signal detection on the surface of pneumococci (Ctrl). Scale bar represents 10 μm. Fluorescence intensity determination visualizes the VWF-bacterial colocalization within the region of interest (ROI 01).

Samples were fixed in 5% formaldehyde and 2% glutaraldehyde in cacodylate buffer, pH 6.9 (0.1 M cacodylate, 0.01 M CaCl_2_, 0.01 M MgCl_2_, 0.09 M sucrose) for 1 h on ice and washed with TE-buffer, pH 7.0 (20 mM Tris/HCl, 1.0 mM EDTA) before dehydrating in a graded series of acetone for 15 min at each step. Samples were then subjected to critical-point drying with liquid CO_2_ (CPD 30, Bal-Tec, Liechtenstein). Dried samples were covered with a gold film (SCD 500, Bal-Tec, Lichtenstein) before examination in a field-emission scanning-electron microscope Zeiss Merlin (Oberkochen, Germany) using the Everhart Thornley SE detector and the SE inlens detector at a 75:25 ratio with an acceleration voltage of 5 kV. Contrast and brightness were adjusted using Adobe Photoshop CS5. Capsule expression of *S. pneumoniae* serotypes shown in Figure 1 was visualized by transmission electron microscopy on ultrathin sections after lysine-acetate-based formaldehyde-glutaraldehyde ruthenium red-osmium fixation procedure (LRR fixation).

For immune labeling of VWF protein HUVEC cells, grown on cover slips and infected with pneumococci, were incubated with anti-VWF antibodies in a 1:75 dilution of the stock solutions in PBS (mixture of polyclonal rabbit and monoclonal mouse antibodies) for 1 h at 37°C, washed with PBS and further incubated with goat anti-mouse and Protein A coated gold-nanoparticles with 15 nm in diameter in a 1:50 dilution of the stock solutions. After incubation for 30 min samples were washed with PBS and TE buffer and critical-point dried. Dried samples were sputter coated with carbon before examination in a Zeiss Merlin (see above) applying the HESE2-detector and inlens SE-detector in a 85:15 ratio with an acceleration voltage of 10 kV.

### *In vivo* Colocalization Studies

Pneumococcus-VWF colocalization studies and detection of bacterial aggregation were carried out by injecting RFP-expressing serotype 35A bacteria in wild type larvae [WT brass PBS 0545, 5 days post-fertilization (dpf)]. For some experiments, the bacteria were pre-incubated with human VWF as described above. After 2 h of cultivation in 30% Danieau at RT, fixation of the larvae was performed by incubation in 4% PFA in PBS over night at 4°C. The formerly described immune fluorescence staining method was applied with minor modifications (Luttge et al., 2012). The detailed injection procedure and immune staining is described in Supplementary Material. For microscopic visualization, the stained larvae were embedded in 1.0% low melting agarose. For each study, a minimum of 12 larvae were used in two independent experiments. All procedures involving zebrafish were carried out according to EU guidelines and German legislation (EU Directive 2010_63, licence number AZ 325.1.53/56.1-TU-BS).

### Dot Spot Overlay and Peptide Array

Enolase protein (rEno) of *S. pneumoniae* serotype 2 (ATCC 11733) was produced by the *E. coli* strain M15 and purified as histidine-tagged fusion protein by imidazole-mediated purification from NiNTA-columns (Macherey-Nagel) using the clone pQE30*eno*. This clone was previously generated by gene insertion into the pQE30 expression plasmid (Qiagen®) (Bergmann et al., 2001). For dot spot binding analyses, rEno or VWF domains A1, A2, and A3 were immobilized onto a nitrocellulose membrane (Whatman). After blocking with 3% bovine serum albumin (Sigma Aldrich) for 1 h at RT, the blot was incubated for 16 h with 2.0 μg/ml VWF. After washing with PBS supplemented with 0.05% Tween 20, detection of VWF-binding signals was performed by incubation with monoclonal VWF-specific mouse antibodies and alkaline peroxidase-labeled secondary antibodies. Signal detection was visualized using BCIP/NBT liquid substrate system Kit (Sigma Aldrich) according to the manufacturer's instructions. Dot spot analysis was repeated three times in independent analyses. Detection of unspecific antibody binding was analyzed by a second membrane without VWF incubation. Control experiments are included in Supplementary Material. Similarly, VWF domains A1, A2, and A3 were immobilized in amount of 0.125 μg up to 1.0 μg and subjected to rEno-overlay using 4.0 μg/ml rEno. As control, 1.0 μg VWF and 1.0 μg rEno, respectively, was additionally immobilized and detection of unspecific antibody signals was conducted with a second membrane after identical probing but without enolase incubation. Immunoblot analysis with anti-enolase antiserum derived from rabbit was performed using alkaline phosphatase (AP)-conjugated secondary antibody (Thermo Fisher Scientific) and corresponding substrate solution (Sigma Aldrich) as described above. After scanning, brightness and contrast of the blots were adjusted using (Adobe Photoshop CS5, version 12.0).

For peptide spot array, the enolase amino acid sequence was divided into 141 overlapping peptides consisting of 15 amino acids each, with an offset of three amino acids. The peptides were synthesized as an array of spots on an amino pegylated cellulose membrane (AIMS Scientific Products GmbH, Germany) (Beutling et al., 2008). For detection of peptides mediating VWF-binding, membranes were treated as described previously using 2.0 μg/ml VWF followed by incubation with an anti-VWF antibody and a peroxidase-labeled secondary antibody. Binding signals were visualized by staining with 1.0 mg/ml 4-chloro-1-naphthol and 0.1% H_2_O_2_. After scanning, contrast of the blots was adjusted using Adobe Photoshop CS5 without deleting any signal information.

The crystal structure of the octameric enolase of the serotype 2 pneumococcus (ATCC 11733, PDB entry 1W6T) was solved previously at 2.0 Å and was used as matrix for visualization of putative VWF binding sites (Ehinger et al., 2004). Molecular visualization of the putative surface located VWF-binding motives was performed with PyMOL.

### Determination of Binding Kinetics

The association and dissociation reactions of human plasma VWF to recombinant Enolase (rEno) were analyzed in the BIAcore optical biosensor (BIAcore T200 system, GE Healthcare, Munich, Germany) using CM5 sensor chips. Covalent immobilization of rEno was performed using a standard amine coupling procedure as described previously (Bergmann et al., 2003). Binding analyses were performed in phosphate buffered saline (pH 7.4) containing 0.05%Tween 20 at 25°C using a flow rate of 10 μl/minute (min). Plasma VWF (Merck Biochemicals) was used as analyte in concentrations of 0.625, 1.25, 2.5, 5.0, and 10.0 μg/ml and VWF domains A1, A2, and A3 were used as analytes in concentrations of 0.0612, 0.125, 0.25, 0.5, and 1.0 μg/ml. The affinity surface was regenerated with 2.0 mM NaOH. Binding to enolase was examined in two (full length VWF) or three (VWF A1 domain) independent kinetic analyses. Calculated K_D_ and Chi^2^-values were analyzed from raw data using the 1:1 Langmuir binding model (mathematical iteration) included in the Biacore T200 evaluation software version 3.0. Representative fitting results and a table with a detailed list of calculated binding parameter including *K*_a_, *K*_d_, *K*_D_, RU_max_, and Chi^2^-values is added to Figures S4A,B and Table S4C.

## Results

### *Streptococcus pneumoniae* Binds Human Plasma-Derived VWF

Cellular infection analyses demonstrated that *S. pneumoniae* adherence to endothelial cells induces the release of high amounts of VWF from Weibel Palade Bodies into the cell media (Luttge et al., 2012). To investigate if this highly adhesive plasma protein might locally support bacterial cell adhesion in blood vessels, we first performed VWF-pneumococci binding studies. Recruitment of plasma-derived, radioactively labeled ^125^I-VWF to the surface of seven different *S. pneumoniae* strains including clinical isolates with different amounts of capsular polysaccharides (CPS) was determined (Figure 1A). Grading of capsule thickness into low (<0.03 μm), middle (0.03–0.5μm) and high (>0.5 μm) encapsulation was defined by computation of capsule diameter according to electron microscopic visualization. While all serotypes exhibited VWF-interaction, the binding activity increased with decreasing capsule diameter (Figure 1A). These data were confirmed by immunofluorescent detection of VWF on the surface of RFP-expressing *S. pneumoniae* serotype 35A, which is a widely used model organism for interaction studies (yellow in overlay Figure 1B, merge, ROI 01). In summary, radioactive binding analysis and immunofluorescence studies demonstrate that various capsulated and non-capsulated *S. pneumoniae* strains and serotypes are able to bind VWF.

### VWF Mediates Bacterial Adherence to Endothelial Cells

**Figure 2:**
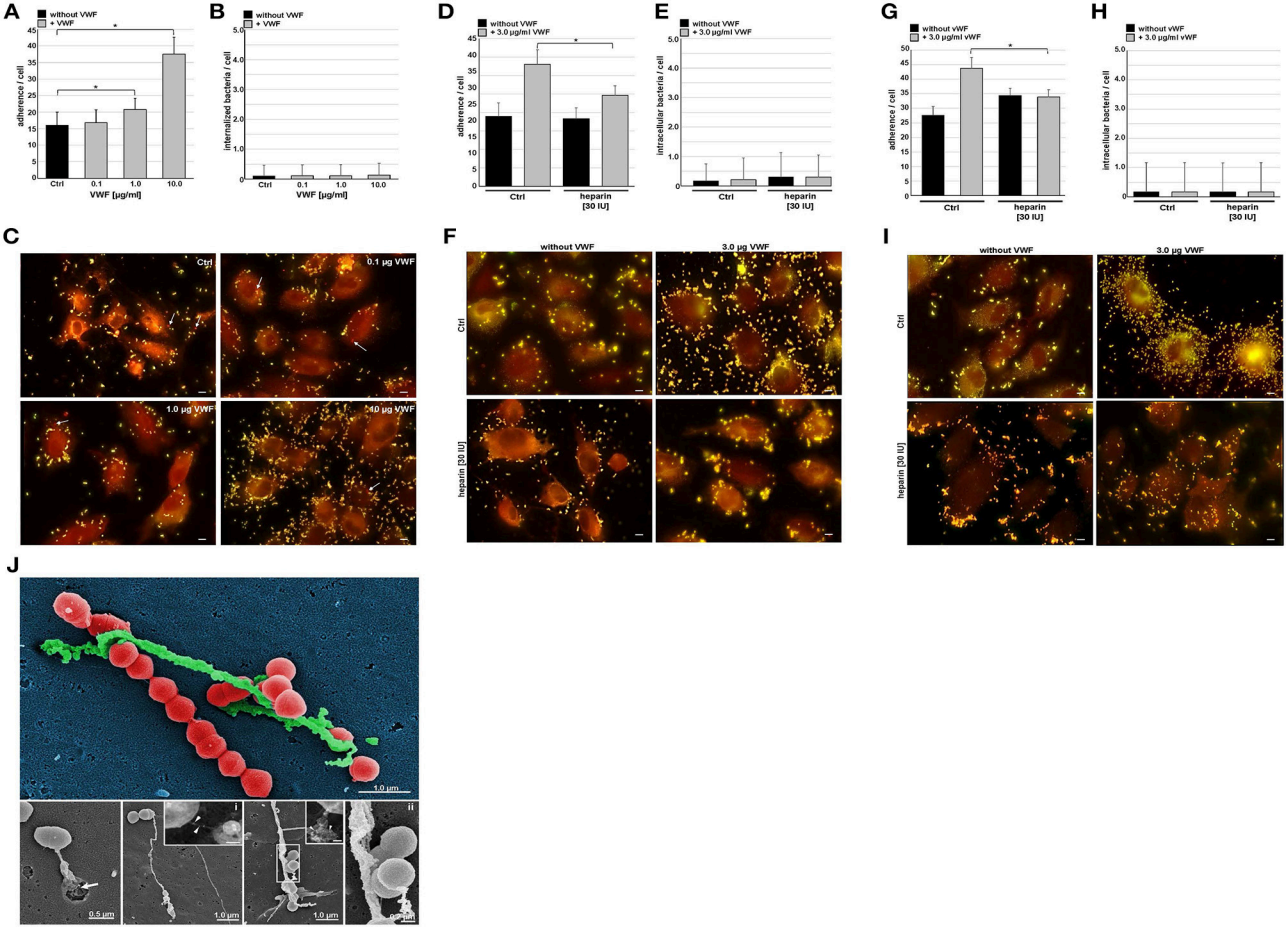
VWF mediates pneumococcal adherence to endothelial cells. Microscopic determination of VWF-mediated bacterial adherence to subconfluently grown endothelial cells (HUVEC) and internalization after immunofluorescence staining with pneumococcus-specific rabbit antibodies followed by Alexa 488 and Alexa 568-conjugated secondary antibodies. HUVEC were pre-incubated with 0.1, 1.0, and 10.0 μg/ml VWF. Three independent assays were conducted in triplicate. After visual control of cell morphology and staining efficiency, number of yellow fluorescent attached bacteria **(A)** and number of red fluorescent internalized bacteria **(B)** were counted for a minimum of 50 endothelial cells in each sample. Representative images are shown in **(C)**. White arrows indicate detection of internalized pneumococci. Scale bar represents 10 μm. Quantification of bacterial adherence **(D)** and internalization **(E)** in cell culture inhibition studies with 3.0 μg/ml VWF in presence of 30 IU heparin. Adherence and internalization were microscopically determined as described above **(F)**. All data shown in Figure 2 represent mean values ± SEM; statistical significance was evaluated by unpaired Student's *t*-test (^*^p < 0.05 was defined as significantly different). Scale bar represents 10 μm. Effect of heparin on extent of VWF-mediated bacterial attachment **(G,I)** and internalization **(H,I)** was further determined after VWF-pre-incubation of bacteria instead of HUVEC. All data shown in Figure 2 represent mean values ± SEM; statistical significance was evaluated by unpaired Student's *t*-test (^*^p < 0.05 was defined as significantly different). Scale bar represents 10 μm. **(J)** Electron microscopic visualization of pneumococcus attachment to protein strings on HUVEC. Samples were subjected to field emission scanning electron microscopic visualization with a Zeiss Merlin FESEM, which illustrates pneumococcal attachment to long protein strings resembling VWF-strings in length and size. Colors have been applied by Adobe Photoshop CS5 v12.0 for emphasizing structural differences. RFP-expressing pneumococci are colorized in red, HMW VWF protein strings appear in green and the HUVEC cell background appears in dark blue. The white arrow in the lower left image points to a diplococcus bound to a VWF protein string, which is released by a secretory pod on the cell surface. The two insets visualize a VWF-specific immune-gold staining of a long, thin VWF-string **(i)** and of a short VWF string **(ii)**. The gold-conjugated particle are marked with white arrow heads. The lower right picture shows a 5-fold magnification of the region marked with a white square. Scale bars of the insets represent 0.2 μm, the other scale bars are depicted in illustrations.

Since pneumococci can recruit plasmatic VWF, we further investigated if this interaction might influence bacterial attachment to the endothelium. We performed cell culture infection studies with HUVEC and serotype 35A pneumococci, which had been pre-incubated with VWF. This serotype is less capsulated and is widely used as reliable model organism in many cell culture infections (Bergmann et al., 2009; Luttge et al., 2012). Serial immunofluorescent staining of non-permeabilized and permeabilized cells was used to distinguish between surface-attached and internalized bacteria, respectively, and revealed an increase in VWF-mediated bacterial attachment depending on the amount of endothelial-bound VWF (Figures 2A,C), whereas VWF did not influence intracellular uptake of pneumococci into HUVEC (Figures 2B,C, marked by white arrows). The A1-domain of VWF exposes a heparin binding site (Rastegar-Lari et al., 2002). Thus, to assess whether heparin competes with VWF binding to HUVEC and in consequence abrogates the VWF-mediated adherence mechanism, infections were also performed in the presence of 30 IU heparin. Indeed, heparin significantly reduced VWF-mediated pneumococcal adherence (Figures 2D,F). Again, VWF-mediated bacterial internalization was not substantially affected by heparin (Figure 2E). Pre-incubation of bacteria with VWF rather doubled bacterial attachment from 28 bacteria per cell to 44/cell (Figures 2G,I). This increase in attachment was significantly inhibited by heparin (Figures 2G,I). In accordance with data for HUVEC pre-incubation with VWF, bacterial internalization rate remained unchanged (Figures 2H,I).

In addition to the immuno fluorescence visualization, Field Emission Scanning Electron microscopy (FESEM) of the HUVEC surface after 2 h of pneumococcal infection impressively visualizes several spots of bacterial attachment to long protein strings (Figure 2J, colorized image and gray scale images). Valentijn and coworkers already described these strings as HMW VWF structures, which are secreted from characteristic secretory pods (Valentijn et al., 2011) (Figure 2J, bottom panel with white arrow). In addition, immuno-labeling identified those strings as VWF protein containing structures (inserts **i** and **ii** in Figure 2J, bound gold-nanoparticles are marked with arrow heads).

### Pneumococci Bind VWF Strings Under Shear Flow Conditions

**Figure 3:**
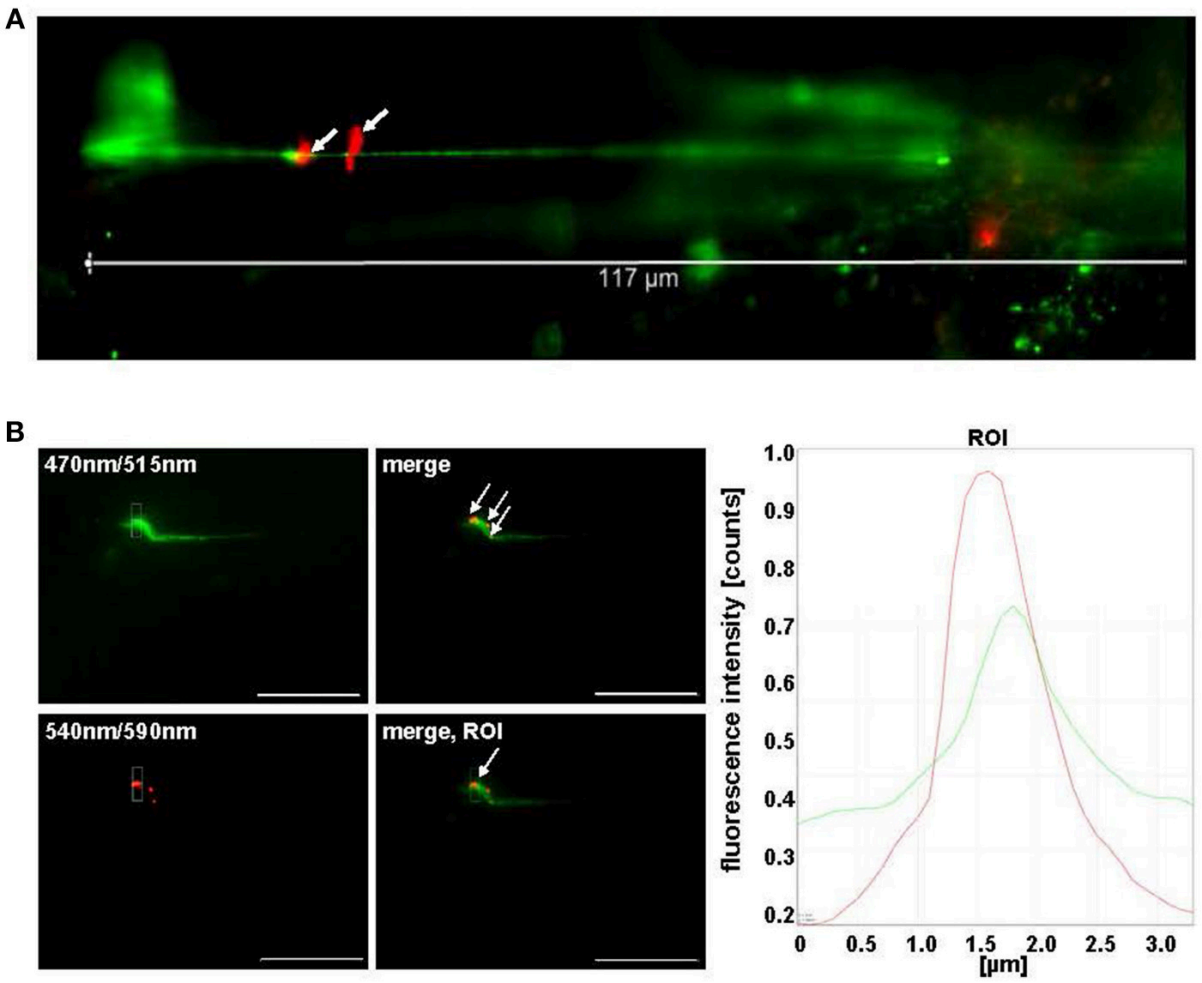
Pneumococci bind to VWF strings generated in continuous flow. **(A)** HMW VWF fiber generation was microscopically quantified after exposing confluently grown HUVEC to shear stress using a microfluidic device (ibidi®) at 10 dyne/cm^2^. FITC-conjugated VWF-specific antibodies detected HMW VWF strings. White arrows point to red fluorescent pneumococci attached to long VWF strings. **(B)** Attachment of RFP-expressing pneumococci to green fluorescent VWF strings was microscopically observed after 30 min in constant flow (white arrows) and was confirmed by software-based evaluation of fluorescence intensities of the defined ROI. Pictures were taken in real time using the fluorescence equipment of a confocal laser scanning microscope (SP8, Leica). Scale bar represents 10 μm.

VWF is a mechanosensitive protein, which alters its structural conformation and its multimerization status upon shear stress generated by the blood flow (Ruggeri, 2001). With the aim to analyse pneumococcus attachment to multimerized VWF strings, a microfluidic approach (ibidi®) was established (Bergmann and Steinert, 2015). This approach enabled the simulation of the situation in the human vascular system, in which VWF strings are generated on endothelial cells under flow conditions. In order to visualize and to quantify pneumococcus attachment to generated VWF strings on endothelial cells, VWF-secretion of confluently grown HUVEC was induced by histamine stimulation at a shear rate of 10 dyne/cm^2^ and RFP expressing serotype 35A pneumococci grown to mid log phase were injected into the flow. The first events of bacterial attachment to VWF strings were detected after 30 min post-injection of RFP-expressing pneumococci to the histamine-stimulated HUVEC in flow (Figures 3A,B, white arrows). On average, pneumococcus attachment was monitored at one out of eight visualized VWF strings after 90 min at constant unidirectional shear flow and was confirmed by histogram overlay showing overlapping fluorescence peaks for VWF and attached pneumococci (Figure 3B). The bacteria remained attached to the VWF strings for at least 25 min at constantly high shear rates. Absolutely no bacterial attachment to VWF strings was detected in the presence of heparin (Figure S7), which strongly suggested that heparin binding sites are required for bacterial attachment to VWF strings in flow. In addition to a recruitment of circulating VWF to the bacterial surface, the microfluidic approach confirmed a tight attachment of pneumococci to VWF strings generated in flow.

### Pneumococci Colocalize With VWF in *Danio rerio* and Form Bacterial Aggregates

**Figure 4:**
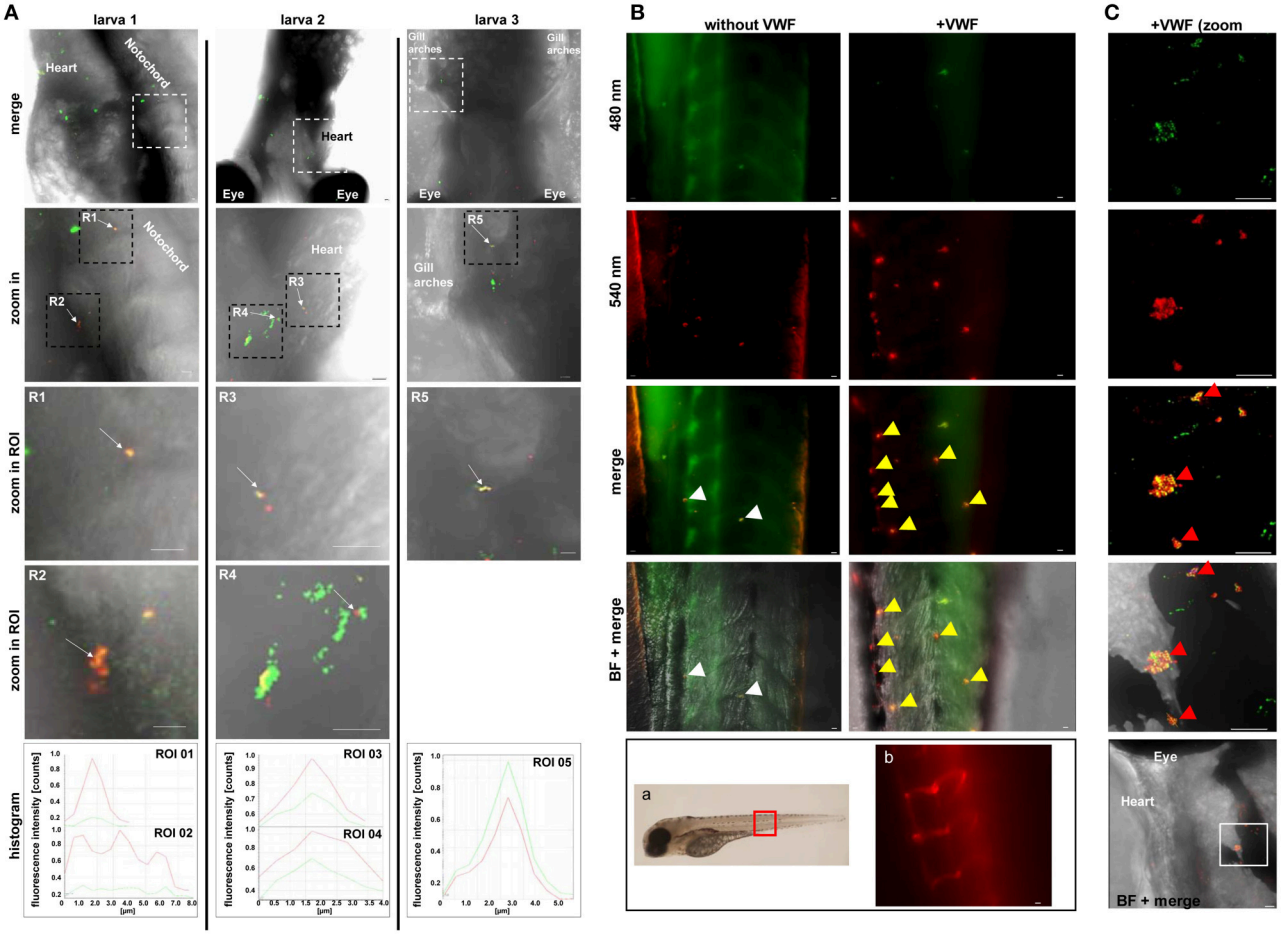
Colocalization of pneumococci with VWF in vascular system of *Danio rerio*. **(A)** Colocalization of red fluorescent pneumococci with zebrafish-derived VWF was microscopically visualized after fixation, embedding, and staining of wild type larvae with VWF-specific rabbit FITC-conjugated antibodies and pneumococcus specific rabbit antibodies followed by goat Alexa 568-conjugated antibodies. Visualization of three representative larvae (no. 1, 2, and 3) is shown after digital zoom in magnification of the region marked by white and subsequently by black squares. All larvae are displayed from ventral side. The white square encircles intersegmental vessels of the trunk muscles in larva 1, the heart vasculature of larva no. 2 and the vessels of the gill arches of larva no. 3. White arrows of R1–R5 point to VWF colocalization with single diplococci or short pneumococcus chains appearing as yellow overlay. Colocalizations within selected regions of interest (zoom in ROI) are confirmed by corresponding overlay histograms. Scale bars represent 10 μm. **(B)** Visualization of RFP-expressing serotype 35A pneumococci in zebrafish larvae without and after pre-incubation of pneumococci with human VWF prior to injection of 600 cfu into the heart chamber. The red square in the zebrafish illustration depicts the region of the zebrafish tail, which is focused for visualization after immunostaining. Using a transgenic zebrafish larva, which expresses red-fluorescing endothelium (FLK1:mCherryCAAX), the position of the main blood vessel and its branching circular microvasculature within the tail region are visualized in b. After 5 h of infection with non-VWF-pre-incubated RFP-expressing pneumococci (without VWF), only single diplococci are detected within the intersegmental blood vessels of the tail region of cross linked larvae after immune staining of VWF (green) (without VWF, merge, white arrows). The yellow appearance in the merged illustration points to the recruitment of zebrafish-derived VWF to the surface of the bacteria. After 5 h of infection with VWF pre-incubated pneumococci, several bacterial aggregates were detected after immunostaining of VWF in green and bacteria in red as yellow clumps of up to 10 μm in size in the vasculature of the zebrafish tail (+VWF, merge and BF + merge, yellow arrows). **(C)** A further representative example of VWF-mediated pneumococcal aggregate formation with the vasculature of an infected zebrafish larva. A zoom in into the zebrafish region in close proximity to the larval heart (white square) visualizes a cluster of pneumococci (+VWF, merge, red arrows). A histogram of fluorescence signal overlaps in the aggregates is added in Figure S3B. Scale bars represent 10 μm.

In order to confirm that VWF can trigger pneumococci aggregation in the vasculature *in vivo*, the *D. rerio* vertebrate model was chosen. This *in vivo* model provides a complete blood environment whose hemostasis and thrombosis mechanisms and factors share high similarity to the human physiology especially in terms of coagulation and fibrinolysis (Hanumanthaiah et al., 2002; Jagadeeswaran, 2005; Ghosh et al., 2012; Weyand and Shavit, 2014), which renders this model suitable to study the role of VWF-pneumococcus interaction in hemostasis. After injection of RFP-expressing serotype 35A pneumococci into the heart chamber of 5 days old wildtype zebrafish, microscopic visualization of histological immunofluorescence staining detected VWF recruitment to circulating diplococcoid pneumococci or short pneumococci chains into various regions of the vasculature and nearby tissue in the larvae (Figure 4A, larvae 1, 2, 3, white arrows). Fluorescence intensity histograms confirmed the localization of VWF-coated pneumococci in intersegmental blood vessels passing through the trunk muscles (larva no. 1), in close proximity to the heart (larva no. 2) and within the gill arches that sustain the vasculature (larva no. 3). The colocalization of VWF with pneumococci circulating in zebrafish larvae demonstrates that the recruitment of VWF to the pneumococcal surface takes place *in vivo*.

To confirm the visualization of bacterial circulation within the fish vasculature, RFP-expressing pneumococci were injected into the heart chamber of larvae of a zebrafish transgenic strain TgCflk1:mcherryCAAX expressing a membrane targeted red fluorescent protein in endothelial cells. Representative images of the vasculature of three larvae (Figure S3A) in addition to videos of the region depicted for larva no.3 are shown in Videos S1–S3. These recordings reaffirm the localization of pneumococci along the endothelium.

With the aim to visualize effects of pneumococcus-VWF-interaction on vascular integrity, bacteria were injected into the heart chamber of zebrafish larvae without VWF and after pre-incubation of RFP-expressing pneumococci with human VWF. Without VWF-pre-incubation of pneumococci, only single diplococcoid bacteria were detected 5 h after zebrafish-infection (Figure 4B, without VWF, merge, white arrows). Since the VWF signal was visualized by green fluorescence after immuno staining with VWF-specific antibodies, the yellow appearance of pneumococci in the merged illustration indicates the recruitment of zebrafish-derived VWF in green to the surface of the red fluorescing bacteria (Figure 4B, without VWF, merge, white arrows). As physiological reference and orientation, the tail region of the zebrafish larvae, which was used for representative microscopic visualization of pneumococci after infection, is marked with a red square (Figure 4Ba). In addition, a fluorescence micrograph of this zebrafish region shows the position of the red fluorescent endothelial vasculature of a transgenic zebrafish (TgCflk1:mcherryCAAX, Figure 4Bb).

After 5 h of larval infection with VWF-pre-incubated pneumococci, VWF-containing bacterial aggregates of up to 10 μm in diameter were detected by immune microscopy within the vasculature of the larval tail region (Figures 4B,C with VWF, merge, yellow arrows). The aggregates line the curved shape of the main blood vessel and appear very often at the bending of vascular branching sites, which display a non-laminal blood flow and a slightly reduced flow speed. Besides the tail region, bacterial aggregates were found to be distributed in a widespread manner and could be visualized for example in the vasculature near the heart or close to the eye (Figure 4C). A histogram of fluorescence signal overlaps in the aggregates is added in Figure S3B. This immune staining supports the idea that VWF recruitment to the pneumococcal surface contributes to bacterial aggregation within the vasculature *in vivo*.

### VWF Binds to the Non-Classical Surface Protein Enolase

**Figure 5:**
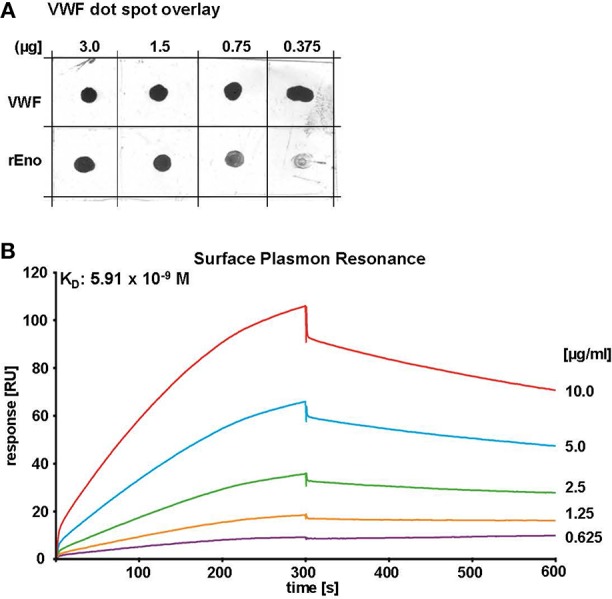
Enolase of *S. pneumoniae* binds VWF derived from human plasma with high affinity. **(A)** VWF dot spot overlay of recombinant enolase protein (rEno) of *S. pneumoniae* and VWF which were immobilized by dot spotting onto nitrocellulose in indicated amounts (VWF was used as positive control). Dose-dependent VWF-binding signals to rEno were detected by VWF-specific antibodies and visualization by chemiluminescence. Antibody controls are shown in Figure S1A. **(B)** Binding kinetics of rEno and plasma-derived VWF were determined by SPR using rEno as immobilized ligand on CM5 Biacore Chips and plasma VWF as analyte in concentrations of 0.625–10 μg/ml. Data evaluation was determined in two independent analyses using a 1:1 Langmuir model and revealed an average dissociation constant *K*_D_ of 5.36 × 10^−9^ M at a Chi^2^-value of 0.665. Kinetic determination was performed with a BIACORE®T200 (GE Healthcare) at a flowrate of 10 μl per min. A representative sensorgram is shown.

After having shown that pneumococci bind to VWF, we strived to identify the interacting bacterial surface protein. In previous studies, we described the major glycolytic enzyme enolase of *S. pneumoniae* as surface-located binding protein for another plasma glycoprotein, namely the host-derived fibrinolysis factor plasminogen (Bergmann et al., 2001, 2003, 2005). Since VWF is also a plasmatic glycoprotein, we investigated if enolase might also exhibit VWF-binding activity. Dot blot screening analyses indeed revealed binding of purified His_6_-tagged pneumococcal enolase (rEno) to VWF in a dose-dependent manner (Figure 5A).

Binding of VWF to immobilized enolase protein was also further biochemically characterized by surface plasmon resonance analysis, which provides the determination of kinetic parameters of the VWF-enolase interaction (SPR, Figures 5B, S4A and Table S4C). At flow rates of 10 μl per minute different amounts of plasma-derived, non-labeled VWF bound to enolase in a dose-dependent manner. Evaluation of binding data using a 1:1 Langmuir model revealed an average dissociation constant (*K*_D_) of 5.36 × 10^−9^ M (Figure 5B), thereby indicating a strong interaction.

### VWF Binds to a Putative Binding Groove of Pneumococcal Enolase

**Figure 6:**
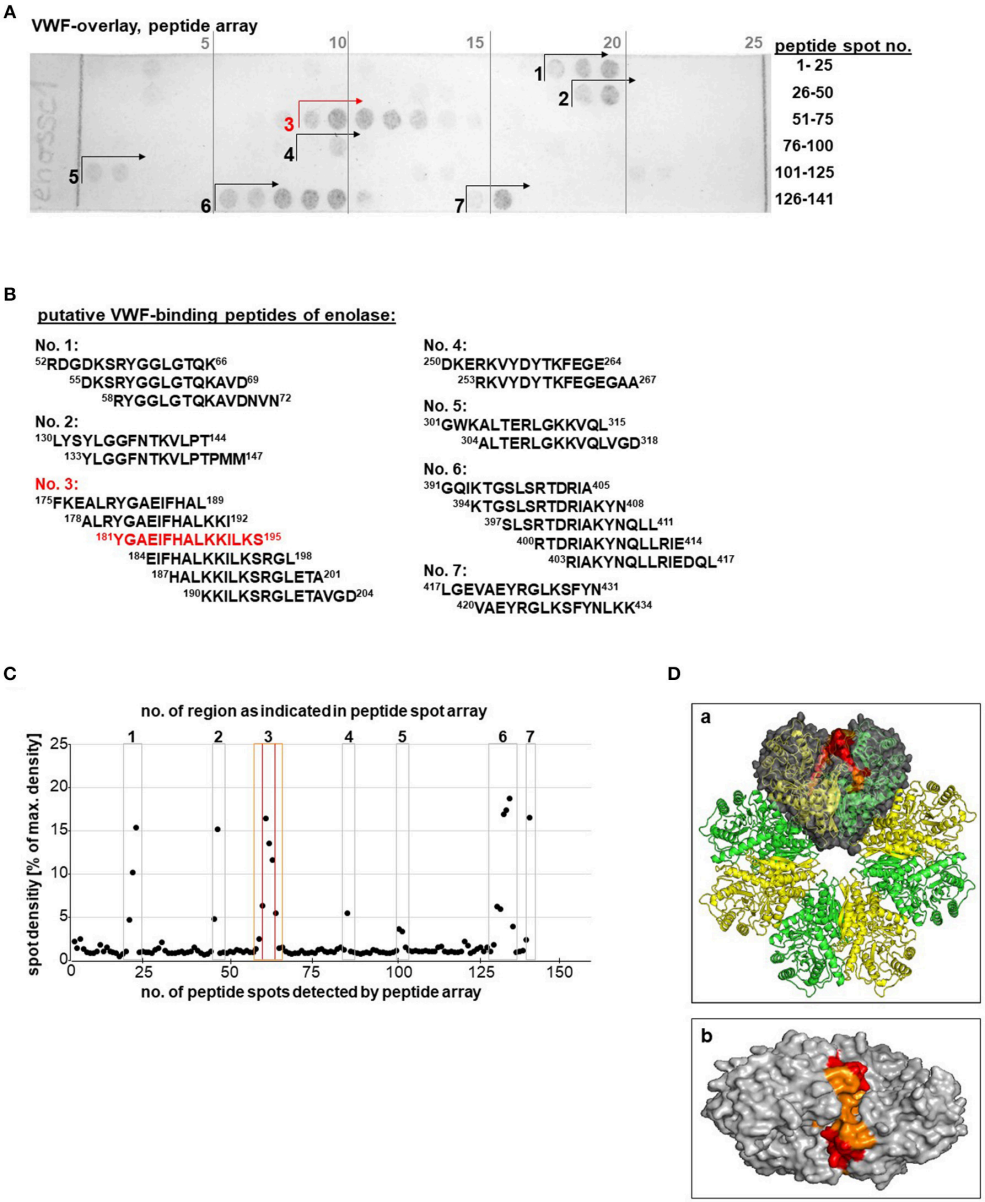
Identification of a putative VWF binding site on the pneumococcal enolase. **(A)** VWF overlay of enolase peptide spot array, representing the whole enolase amino acid sequence, identified up to seven regions displaying VWF-binding activity. Antibody controls are shown in Figure S1B. **(B)** Table of peptide sequences of all peptide spots showing VWF-binding activity. The strongest VWF-binding signal shows peptide “^181^YGAEIFHALKKILKS^195^” of region no. 3 (marked in red). **(C)** Densitometric quantification of signal intensity. The seven regions with positive signals are numbered and marked with squares. Peptide region Nr. 3 is located on the enolase molecule surface and the core peptide is marked in red and the adjacent peptide spots are marked in orange. **(D)** Localization of the putative VWF-binding pocket within the octameric enolase molecule depicts alternating enolase monomers colored in yellow and green; the VWF-binding site is highlighted within one of the four dimers colored in gray **(a)**. The whole VWF-binding peptide region no. 3 representing aa 181–195 is marked in orange and the core peptide composed of aa 181–195 is marked in red. Top view of the VWF binding groove of a dimer is shown in **(b)**. Additional views are added in Figure S5. Structure visualization was calculated using PyMOL.

As surface displayed moonlighting molecule, the enolase elicits a couple of adhesive functions which are mediated via different binding domains on the molecule surface (Fulde and Bergmann, 2017). With the aim to identify putative VWF-binding domains of the pneumococcal enolase, the whole enolase amino acid (aa) sequence was scanned by peptide spot analysis with 15mer overlapping peptides (Figure 6A). The VWF overlay assay revealed positive and VWF-specific signals for 21 peptides comprising seven putative protein binding sites (Figure 6B). Only marginal unspecific background binding of the applied antibodies is detected without VWF incubation (Figure S1B).

Based on a densitometric quantification of spot intensity, only the peptide regions 1, 2, 3, 6, and 7 mediated strong VWF interaction signals (Figure 6C). The structural localization of all seven regions within the three-dimensional enolase molecule structure formerly solved by Ehinger and colleagues (Ehinger et al., 2004) revealed that six of the seven putative binding regions are mostly hidden within the molecule. Only the protein region no.3 (aa 189–219, Figure 6D; marked in orange) with the strongest VWF-binding core peptide ^181^YGAEIFHALKKILKS^195^ (Figure 6D; marked in red), is located on the molecule's surface at the interface of two adjacent enolase monomers (Figure 6Da), thereby possibly forming a long putative VWF-binding pocket on each of the four enolase dimers (Figure 6Db). The grooved shape of the binding pocket formed by the VWF-interacting peptides colored in red and orange is also depicted in the top view (Figure 6Db and Figure S5). Results of the peptide array in combination with structural localization provide a first hint of a putative VWF-binding site.

### The A1 Domain of VWF Serves as Enolase Binding Site

**Figure 7:**
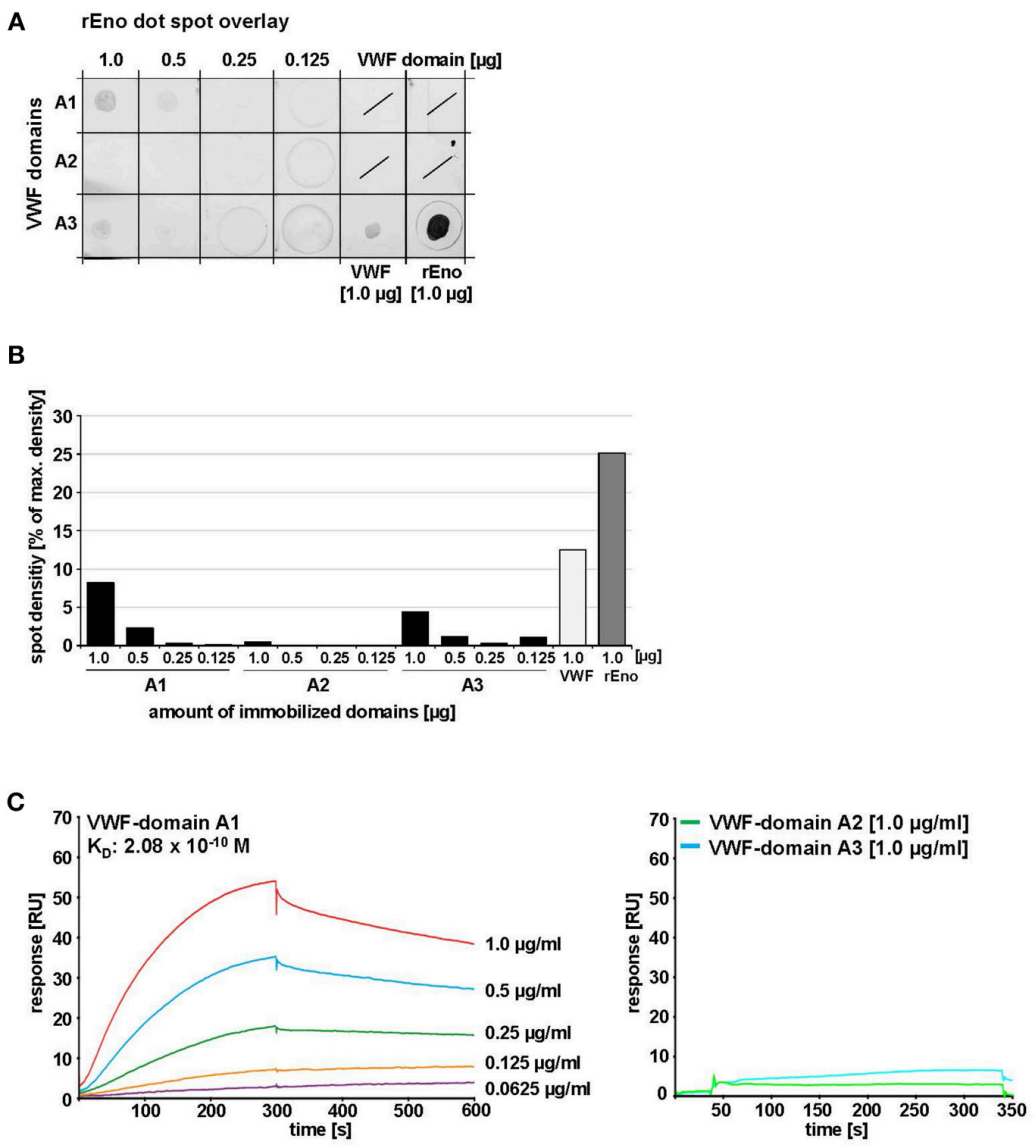
Identification of VWF domains interacting with *S. pneumoniae* enolase. **(A)** Enolase protein overlay of VWF domains A1, A2, and A3 after immobilization of these domains by protein dot spotting on nitrocellulose at indicated amounts (1.0, 0.5, 0.25, and 0.125 μg). 1.0 μg VWF protein was immobilized as positive binding control and 1.0 μg rEno as antibody control. Protein overlay was performed with 1.0 μg/ml rEno and enolase-specific antibodies. Antibody overlay controls are shown in Figure S2A. **(B)** Densitometric analysis was performed with ImageJ version 1.49v after subtraction of antibody background. Mean density values are displayed as percent in relation to maximum density. **(C)** SPR analysis of immobilized rEno on Biacore CM5 sensor chips confirmed binding of VWF domain A1 to immobilized rEno, whereas no specific binding of VWF domains A2 and A3 to immobilized enolase was detected. Kinetic analyses revealed an average *K*_D_ of 2.08 × 10^−10^ M with a Chi^2^-value of 2.4 for the interaction of rENo with VWF domain A1 using a Langmuir 1:1 model. Three independent binding analyses were performed with a BIACORE®T200 (GE Healthcare) at a flowrate of 10 μl/min after immobilization of 1,700 RU rEno. A representative sensorgram is shown for VWF domain A1. A coomassie stain confirming protein integrity of the A domains is added in Figure S2B.

Depending on the location of the binding site, the enolase might interfere with crucial VWF functions. The VWF A domains are involved in binding of a variety of protein partners, thereby mediating crucial functions of VWF in hemostasis (Springer, 2014). To identify the VWF binding domain for the enolase we first performed a dot spot overlay using purified single VWF A domains. Densitometric analysis indicated that the strongest signal for enolase binding was detected for A1 (Figures 7A,B). A3 gave only a marginal signal while A2 exhibited no binding (Figures 7A,B). For antibody negative controls, the positive control with full-length VWF, and the SDS-PAGE analysis of A domain peptides please refer to Figures S2A,B. Evaluation of kinetic parameters by SPR studies confirmed the high affinity interaction of immobilized enolase with the VWF A1 domain with an average K_D_ of up to 2.08 × 10^−10^ M in the 1:1 Langmuir model (Figures 7C, S4B and Table S4C). In contrast, no specific interaction was detected to VWF A2- and A3 domains (Figure 7C). This data identified the A1 domain of VWF as binding site of pneumococcus enolase.

## Discussion

This report for the first time describes both, binding of *S. pneumoniae* to globular as well as shear-elongated VWF and that this interaction promotes bacterial adherence to endothelial cells *in vitro* and *in vivo*. Previously, a direct VWF binding has only been reported for two *Staphylococcus* species, *S. aureus*, and *S. lugdunensis* (Herrmann et al., 1997; Nilsson et al., 2004). Similar to encapsulated *Staphylococcus* strains, serotype-dependent differences in capsule expression of pneumococci are affecting the VWF binding activity. Of note, all tested pneumococcus serotypes bound VWF. *S. pneumoniae* is a pathobiont of the human respiratory surfaces and in addition to a symptomless colonization, pneumococci cause local infections and life threatening diseases, such as pneumonia, meningitis and septicemia (Cartwright, 2002; Weiser et al., 2018). In order to facilitate the colonization of mucosal surfaces, pneumococci produce a broad repertoire of cell surface proteins which either mediate direct adherence to eukaryotic cell surface receptors or subvert adhesive matrix molecules and plasma-proteins, such as vitronectin, fibronectin, and plasminogen as molecular bridges for bacterial adherence (Bergmann and Hammerschmidt, 2006; Voss et al., 2012; Weiser et al., 2018). Our cell culture infection analyses with pneumococci showed a dose-dependent VWF-mediated adherence to HUVECs, which prompted us to designate VWF as a novel adhesion cofactor for pneumococcal attachment to the endothelium.

Pneumococci also adhere to endothelial cell surface anchored VWF strings in shear flow of 10 dyne/cm^2^. Of note, pneumococcal attachment to VWF strings is a reproducibly monitored event in our assays, which is also characterized by substantial stability. Moreover, *S. pneumoniae* is the most frequent cause of community acquired pneumonia (CAP), which can progress to a septicemia due to systemic dissemination (Jain et al., 2015). The strong and shear flow resistant attachment of pneumococci to VWF strings generated on activated endothelium might contribute to the severe infection outcome, since the attached pathogen might be protected against mechanical clearance of the blood flow. In this respect, the pneumococcus-VWF interaction may directly contribute to the pathogenesis of severe pneumococcus infections.

We further identified the pneumococcal enolase as a novel VWF binding protein and it is the first that has been described for pneumococci. Only three bacterial VWF-binding proteins have been identified so far; in 2000 (Hartleib et al., 2000) and in 2002 (Bjerketorp et al., 2002), *S. aureus* protein A (SPA) and protein vWbp. The third was found by Nilsson and coworker and named VWF-binding protein (vWbl), which is expressed by *S. lugdunensis* strains (Nilsson et al., 2004). In contrast to the shear stress-dependent VWF interaction with SPA (Pappelbaum et al., 2013), enolase also interacts with soluble plasma VWF. Biochemical characterization of VWF binding kinetics has so far only been reported for SPA by SPR studies demonstrating a *K*_D_ of 1.49 × 10^−8^ mol (Hartleib et al., 2000). We determined *K*_D_ values of similar order of magnitude by SPR (5.36 × 10^−9^ mol), pointing to the high physiological relevance of VWF-binding to pneumococcal enolase. The enolase is a major glycolytic enzyme, which converts 2-phosphoglyceric acid into phosphoenolpyruvate and immune electron microscopic visualization confirmed that it is ubiquitously expressed and surface exposed by all *S. pneumoniae* strains irrespective of serotype and capsule expression (Bergmann et al., 2001). Genetic deletion of the enolase gene is lethal due to the lack of any compensatory energy pathways; therefore, knock-out experiments are impossible. Moreover, the hydrolytic activity of the enolase depends on an accurately folded octameric structure which renders the protein function highly sensitive toward site-specific mutagenesis resulting in single point mutations. Since the putative VWF binding site is located within a structurally important interdimer region, experimental methods for binding site reaffirmation are limited.

In former studies, we characterized the enolase as a surface displayed plasminogen binding protein, which promotes bacteria-driven fibrinolysis (Bergmann et al., 2001, 2003, 2005; Fulde et al., 2013). The plasminogen binding site (^248^FYDKERKVY^256^) is localized at a surface exposed loop in spatial distance to the identified VWF binding pockets formed by the sequence ^181^YGAEIFHALKKILKS^195^, indicating that the enolase might be able to interact with both plasma proteins. The consequences of VWF binding for plasminogen-mediated fibrinolytic activity of pneumococci and *vice versa* is a matter of further in depth studies.

Interestingly, another group revealed that virulence-associated functions of streptococcal and staphylococcal enolases, such as plasminogen binding, are also exhibited by enolases from commensal lactobacilli (Antikainen et al., 2007). In line with this report, we also described an interaction of the bifidobacterial enolase with plasminogen in former studies (Candela et al., 2009). Similar to lactobacilli, bifidobacteria are considered as non-pathogenic commensals and colonize the human intestine. Despite the fact that the catalytic domain of enolases and their function as plasminogen binding protein are highly conserved among different bacterial species, an amino acid sequence comparison of putative VWF-binding sites between enolases from *S. pneumoniae, S. aureus, Lactobacillus plantarum, E. coli*, and *Bifidobacterium lactis* revealed significant differences between pathogenic species and commensals (refer to Table S6). It has been suggested that the plasminogen binding activity of *Bifidobacteria* might improve their long-term settlement within the mucus layer of the gut enterocytes (Candela et al., 2009). Similar to the subversion of plasminogen for bacterial attachment to host tissues, the VWF-pneumococcus interaction promotes the endothelial cell adherence and provides a first spot of settlement for circulating cocci in the blood stream. This might facilitate the activation of further infection steps, which are depending on the specific virulence equipment of each bacterial species. In this respect, the specific sequence differences in VWF-binding sites of bacterial enolases might indicate that the VWF binding via enolase contributes to the infection process and characterizes the enolase-VWF interaction as new virulence trait of pneumococci.

With the aim to obtain information about a possible functional interference of the enolase-VWF interaction with crucial VWF activities, we analyzed the VWF binding sites, which are targeted by enolase. As VWF interaction site for enolase of *S. pneumoniae*, we identified the VWF A1 domain. It has been demonstrated that the shear forces within the blood flow stretch the VWF molecule, thus exposing functional binding sites of the A1 for platelets (Springer, 2014; Lof et al., 2017). The VWF A1 domain provides binding sites not only for the platelet glycoprotein Ib and heparin, but also for collagen types I, IV, and VI and to some extent also type III (Rastegar-Lari et al., 2002; Schneider et al., 2007; Flood et al., 2015). In accordance to our data, the A1 domain was identified as binding site for *S. aureus*, although solely at high shear flow (Pappelbaum et al., 2013). Our binding studies clearly confirmed that a molecular stretching of VWF is not necessary to enable an interaction with enolase or alive pneumococci. This is a remarkable result, since the multidomain glycoprotein VWF circulates as a globular molecule in the human vasculature at a concentration range between 6.0 and 14.0 μg/ml (Spiel et al., 2008). Pathogenesis of bacterial infections, such as the infective endocarditis involves the enhanced secretion of VWF, which elicits crucial functions in platelet adhesion and thrombus formation at endothelial damage sites (Springer, 2014). VWF is released in high amounts during stress, inflammation, sepsis, and cardiovascular diseases and is also regarded as an indicator of vascular dysfunction (Spiel et al., 2008; Springer, 2014). As demonstrated by us in previous studies, luminal VWF secretion from WPB of HUVEC and human lung EC's is significantly increased in response to pneumococcal adherence (Luttge et al., 2012). This increase in VWF secretion might result in higher concentrations of VWF in plasma, which in fact is also associated with an increased risk of myocardial infarction, ischemic stroke, and arterial thrombosis (Sakai et al., 2000; Gragnano et al., 2017). Moreover, Kawecki and colleagues published a comprehensive review about case reports and animal studies demonstrating that inflammation additionally leads to local inhibition of ADAMTS13-mediated VWF cleavage and to a dysbalanced VWF:ADAMTS13 ratio, thereby promoting thrombosis and disseminated intravascular coagulation (Spiel et al., 2008; Kawecki et al., 2017). All these former reports strongly imply a direct contribution between the pneumococcus-VWF interaction and the cardiovascular pathophysiology associated with severe systemic infections. In regard of these coherences, we wanted to analyse the interaction of pneumococci with VWF in a complex living system and performed infection analyses with *Danio rerio* larvae as *in vivo* model of a complete blood environment. It should be noted that the zebrafish endothelium shares high morphologic and functional similarity to the human endothelial tissue (Kamei et al., 2006) and several publications describe the presence of intrinsically and extrinsically triggered coagulation pathways in *D. rerio* (Hanumanthaiah et al., 2002; Weyand and Shavit, 2014). The factors involved in hemostasis, such as VWF share identical functionalities with human clotting factors and also a high degree of protein sequence similarities (Hanumanthaiah et al., 2002; Weyand and Shavit, 2014). This gives solid reason for the extended use of this model for studying the interaction of hemostatic proteins and its suitability for addressing our scientific questions. Additionally, during the last decade, many reports describe infection studies of larval and adult zebrafish with different streptococcal species and present the zebrafish as valuable *in vivo* model for streptococcal and pneumococcal virulence (Phelps et al., 2009; Patterson et al., 2012; Rounioja et al., 2012; Borst et al., 2013; Saralahti et al., 2014; Kim et al., 2015; Saralahti and Rämet, 2015; Jim et al., 2016; Membrebe et al., 2016; Zaccaria et al., 2016; Alves-Barroco et al., 2018). As further advantage, the transparency of the zebrafish facilitates microscopic monitoring. Our fluorescence microscopic visualization results of zebrafish infections demonstrate the attachment of pneumococci at endothelial vessel walls and confirm the recruitment of endogenous zebrafish-VWF to the surface of pneumococci circulating in zebrafish larvae. In line with these observations, a further most important finding is the VWF-mediated bacterial aggregation of up to 10 μm in diameter, which we only detected in bacterial *in vivo* infection of alive zebrafish larvae. The VWF-mediated bacterial aggregate formation in the zebrafish vasculature might cause a partial or a complete occlusion of the larval microvasculature and might contribute to vascular dysfunction, which is known to be implicated in several different severe cardiovascular complications. For example, some case reports highlight the relevance of *S. pneumoniae* infection as cause of uncommon but repeatedly diagnosed abdominal infectious aortitis (Maclennan et al., 1997; Bronze et al., 1999; Cartery et al., 2011). This diagnosis is often associated with a high mortality rate due to non-specific disease symptoms which result in a long diagnosis delay (Postema et al., 2011). In addition, post-infectious vasculitis is described as complication occurring after pneumococcal meningitis (Lucas et al., 2016), which might also induce ischemic cerebrovascular accidents (Khardenavis et al., 2017). According to a study based on 87 cases of pneumococcus meningitis, Kastenbauer and Pfister calculated a rate of 21.8% arterial cerebrovascular complications post *S. pneumoniae* meningitis (Kastenbauer and Pfister, 2003). A combination of single photon emission computed tomography (SPECT), cerebral angiography and conventional computer tomography of 14 adults monitored abnormalities in regional cerebral blood flow as frequent findings in patients suffering from bacterial meningitis. These abnormalities in blood flow might directly contribute to inflammation-mediated increase in VWF-concentration and subsequent coagulation processes (Förderreuther et al., 1992). Moreover, representing a life threatening disease, CAP has been identified as an independent risk factor for up to one third of CAP patients for the development of major adverse cardiac events (MACE) (Musher et al., 2007; Corrales-Medina et al., 2015; Rae et al., 2016). In this regard, the pneumococcus-VWF interaction might contribute to two further proposed virulence mechanisms, which are supposed to be involved in induction of cardiovascular complications upon pneumococcus infection. First, cardiac tissue analyses of a non-human primate model of pneumococcal pneumonia indicated that *S. pneumoniae* invades the myocardium and induces cardiac injury with necroptosis and apoptosis (Reyes et al., 2017). This study is linked to a former publication by Brown and colleges in 2014, which demonstrated that a direct pneumococcal invasion of the heart is accompanied with the induction of bacteria-filled microlesions within the myocardium, thereby inducing cardiomyocyte death and disruption of cardiac contractility (Brown et al., 2014). Second, Alhamdi and colleges reported a contribution of pneumolysin-induced myocardial injury to life-threatening acute cardiac complications as measured by elevated cardiac troponin concentrations (Alhamdi et al., 2015). The impact of pneumolysin on myocardial damage was further specified in mouse infection studies, which demonstrated pneumolysin-mediated necroptosis within the cardiac tissue following pneumococcal myocardial invasion (Gilley et al., 2017). The findings are in line with our former studies showing that VWF secretion of human endothelial cells is triggered by pneumolysin (Luttge et al., 2012). In the same study we demonstrate that attachment of pneumolysin-negative pneumococcus strains to endothelium induces VWF secretion to similar levels as pneumolysin-producing strains, indicating that pneumolysin is strongly promoting but not exclusively required to induce cardiovascular deficiencies (Luttge et al., 2012).

Evaluating our data under consideration of the published context, we suggested that pneumococcus-derived cardiovascular injury occurs via at least three different pathomechanisms: (i) pneumococcal invasion of myocardial cells, (ii) cytolytic activity of pneumolysin, and (iii) VWF-mediated cardiovascular occlusion. These three pathomechanisms might cause the reported cardiac injury as complication of pneumococcal infection in synergistic manner.

In summary, our data demonstrate the recruitment of VWF to the bacterial surface and the use of VWF-strings as endothelial anchor sites for the cocci in blood flow. Our data provide strong evidence that in addition to the VWF-mediated bacterial attachment to the vascular endothelium, this interaction induces the formation of bacterial aggregates leading to distributed vascular occlusions. Since vascular occlusion has been reported as a major cause for cardiovascular diseases, our data provide a possible link between the pneumococcus-VWF binding to the severe cardiovascular complications described in contribution to systemic pneumococcus infections.

## Data Availability

The datasets generated for this study can be found in RCSB PDB, PDB-1W6T.

## Author Contributions

HJ performed and designed experiments. SB designed experiments and wrote the manuscript. I-KB established flow cultivation. KL performed HUVEC infections. RK designed and established zebrafish experiments. RS provided proteins and supported experimental design. TO, MB, and GK performed protein purification and supported experimental design. MR performed electron microscopic studies. TK performed Biacore. GL assisted at zebrafish infections. RF and WT prepared the synthesized peptide arrays. MF performed experiments and critically reviewed the manuscript. MS and SH provided discussion and critically reviewed the manuscript.

### Conflict of Interest Statement

The authors declare that the research was conducted in the absence of any commercial or financial relationships that could be construed as a potential conflict of interest.
