# Supplementary material for: Von Willebrand Factor Mediates Pneumococcal Aggregation and Adhesion in Blood Flow
Source: Front Microbiol. 2019 Mar 26;10:511. doi: 10.3389/fmicb.2019.00511 (PMC6443961; doi:10.3389/fmicb.2019.00511)
Supplement: Supplementary file 1 [file Table_1.pdf]

Supplementary Table S1

Table S1. Data of surface plasmon resonance kinetics fitted to the 1:1 Langmuir binding model.

Analyte, kinetic	k_a [1/ms]	k_d [1/s]	K_D [M]	R_{max} [RU]	Chi^2
**vWF, Kinetic 1**	2.55×10^{5}	1.24×10^{-3}	4.85×10^{-9}	123.6	1.53
**vWF, Kinetic 2**	2.37×10^{5}	1.40×10^{-3}	5.91×10^{-10}	122.4	1.53
**A1, Kinetic 1**	2.22×10^{6}	1.30×10^{-3}	5.85×10^{-10}	60.2	4.01
**A1, Kinetic 2**	5.97×10^{5}	1.26×10^{-3}	2.184×10^{-9}	60.17	1.79
**A1, Kinetic 3**	9.77×10^{5}	1.75×10^{-3}	1.79×10^{-9}	57.09	1.4
