# Supplementary material for: Von Willebrand Factor Mediates Pneumococcal Aggregation and Adhesion in Blood Flow
Source: Front Microbiol. 2019 Mar 26;10:511. doi: 10.3389/fmicb.2019.00511 (PMC6443961; doi:10.3389/fmicb.2019.00511)
Supplement: Supplementary file 2 [file Table_2.pdf]

Supplementary Table S2

Table S2. Amino acid sequence comparison of the putative VWF binding site from different pathogenic bacteria and commensals.

	Acc. No. (UniProtKB)	identities [protein] in %	peptide sequence of putative VWF-binding site
S. pneumoniae **serotype 2 (D39)**	Q04KG2 (ENO_STRP2)		^{185}G A P T F K E A L R Y G A E I F H A L K K I L K S R G L E T A V G D E G G F^{223}
S. pneumoniae **(R6)**	Q8DPS0 (ENO_STRR6	**100**	. . . .
S. aureus **(NCTC 8325)**	Q2G028 (ENO_STAA8)	**94**	. . T . . . . S . . W . T . . . . N . . S . . S K . . . .
L. plantarum **(CRL 1506)**	KRU20356.1	**50**	E R T S . R D G F E K I V N T Y . T . . . V I E D A . Y I . G L . . . . . .
B. animalis **subsp.** ***lactis*** **(AD011)**	B8DTI9 (ENO_BIFA0)	**56**	. F Q . Y S . . . Q A . V . V Y . T . . N V . . K Q . . A . G L . . . . . .
E. coli **(K12)**	P0A6P9 (ENO_ECOLI	**59**	. . K . V . . . I . M . S . V . . H . A . V . . A K . M N . . . . Y
