# Supplementary material for: Von Willebrand Factor Mediates Pneumococcal Aggregation and Adhesion in Blood Flow
Source: Front Microbiol. 2019 Mar 26;10:511. doi: 10.3389/fmicb.2019.00511 (PMC6443961; doi:10.3389/fmicb.2019.00511)
Supplement: Supplementary file 6 [file Data_Sheet_1.pdf]

SUPPLEMENTAL MATERIAL

METHODS

Cultivation of endothelial cells

Primary human umbilical vein endothelial cells (HUVEC) (pooled donors) were from Promocell and cultivated in Endothelial Cell Growth Medium (ECGM, Promocell) supplemented with fetal calf serum (FCS, PAA) and a supplement kit from Promocell including epidermal growth factor, basic fibroblast growth factor, heparin and hydrocortisone to sub confluency at 37°C and 5.0% CO_2. The primary cells were used up to passage 5 at maximum in order to maintain a high status of differentiation. For cryo-conservation, the ECGM medium was supplemented with 20% FCS and 10% DMSO (Sigma Aldrich). Subcultivation was performed according to the manufacturer´s instructions with Accutase (Bioscience). Proteolysis was stopped by adding 2 ml ECGM for neutralization.

In vitro cell culture infection

Cell culture infection analyses have been performed according to a standard infection procedure described previously in Bergmann et al. 2001[19]. 2 x 10^5 HUVEC were seeded on glass cover slips placed in a 24 well plate and incubated for 48 h at 37°C and 5.0% CO_2 in ECGM cell culture medium. Prior to infection, HUVEC were incubated for 1 h at 37°C and 5.0% with different concentrations of VWF (0, 0.1, 1.0, and 10.0 µg/ml VWF). Non-bound VWF was removed by exchanging the medium three times with ECGM. *S. pneumoniae* serotype 35A bacteria were grown to mid log phase in THY, washed and photometrically adjusted to the infection dose of 6 x 10^6 bacteria (multiplicity of infection of 10). The cell culture medium was exchanged with basal medium lacking all supplements (ECBM). After 3 h of bacterial

incubation, the infected cells were washed six times with PBS and incubated for another hour prior to cross linking with 3.0% PFA in PBS for immunofluorescence microscopy. Differential immunofluorescence labeling enabled discrimination between attached and internalized bacteria as reported by Lüttge and coworker in 2012[11]. Pneumococcus-detecting polyclonal antiserum generated in rabbit was used in combination with the Alexa 488-conjugated and Alexa 568-conjugated anti-rabbit antibodies. Globular and multimerized fibrillary VWF was detected with monoclonal mouse VWF-specific antibodies followed by Alexa 488 conjugated, or FITC conjugated antibodies. Attached bacteria appear yellow in the overlay images. In some samples, VWF-secretion was stimulated by supplementation of 1.0 mM histamine. For inhibition studies, either HUVEC or bacteria were incubated for 1 h at 37°C and 5.0% CO_2 in ECBM cell culture medium with 30 injection units (IU) heparin in combination with 3 µg/ml VWF. After washing with 500 µl ECBM, HUVEC were incubated with 1.2 x 10^8 bacteria per ml. Following preincubation of 1 x 10^9 bacteria with inhibitors, bacteria were washed with PBS and photometrically adjusted for cell infection to 1.2 x 10^8 bacteria/ml. As control, cells were incubated with bacteria alone or with VWF-coated bacteria without inhibitor substance. For control of inoculum, bacteria were plated in a series of 1:10 dilution on THY- agar. The assays were only evaluated if the inoculum control confirmed the adjusted amount of bacteria.

Flow cultivation and infection

Flow cultivation was performed using the microfluidic system of ibidi® (Munich, Germany) according to the recommendations of the manufacturer. For gelatin coating, 0.4 mm µ-Slides (µ-Slide $I^{0.4}$ Luer, ibidi®) were incubated with 2% porcine gelatine (Sigma Aldrich, Munich, Germany) for 1 h at 37°C. The slides were rinsed with 1 ml PBS and 1 x 10^6 HUVEC were seeded and incubated for 2h at 37°C and 5% CO_2. After the cells were attached to the slide, a continuous medium flow of 5 dyne/cm^2 for the initial 30 min followed by 10 dyne/cm^2 for 48 h was applied at 37°C and 5% CO_2 using a perfusion set of 1.6 mm in diameter and 50 cm in

length. Infection analysis was conducted after the cells reached tight confluence and a high differentiation status. Prior to incubation for up to 3 h with 1 x 10^8 bacteria/ml RFP-expressing bacteria, the cells were stimulated with 1.0 mM histamine for induction of VWF secretion. After immunodetection with 1.3 µg/ml FITC-conjugated goat anti human VWF-specific antibody (Abcam), generation of VWF strings and bacterial adherence was microscopically visualized in real time and documented by snap shots at different time points for up to 3 h. For visualization of VWF factor strings, a FITC-conjugated, goat anti human anti-VWF antibody was applied. For quantification of VWF strings and bacterial adherence, 30 representative fields of view containing ~10 endothelial cells each were optically analyzed. All experiments were performed in at least three independent assays, each in triplicate and the data were expressed as mean standard deviation. Statistical significances were analyzed by the ANOVA one –factorial test in case of the infection analyses with different VWF concentrations and the two-factorial variance analysis in case of the heparin-inhibition analysis followed by a Post Hoc two-tailed unpaired sample test for detailed statistical comparison. *P*-values of < 0.05 were considered to be statistically significant.

In vivo colocalization studies

For visualization of pneumococci circulating in vasculature, larvae of 12 transgenic zebrafish (5 days post fertilization, 5dpf) expressing red fluorescent protein on the endothelium (TgCflk1:mCherryCAAX) were stunned for 4 min with 0.2% Tricaine (Ethyl 3-amino benzoate methanesulfonic Acid, Sigma Aldrich) in 30% (v/v) Danieau containing 2.9 M NaCl, 60 mM $Ca(NO_3)_2$ x 4 H_2O, 40 mM $MgSO_4$, 70 mM KCl, and 0.5 M HEPES. The stunned larvae were placed in an upside down position in a 35 mm dish (ibidi®) and were covered with 1% low melting agarose (Sigma Aldrich). After cultivation of RFP-expressing bacteria as described above, 8 x 10^8 bacteria were resuspended in 10 µl PBS containing 1 µl phenol-red solution

(Sigma Aldrich) for monitoring injection efficiency. For the last experimental set up, 3 x 10^9 bacteria were incubated with 10 µg/ml human VWF for 1 h at 37°C. After three washing steps, the bacteria were adjusted to 2 x 10^9 bacteria per ml. A volume of 0.5 µl bacterial solution with 600 cfu bacteria was injected at 150 hPa injection pressure and at 50 hPa compression pressure into the heart chamber of each larva with a FemtoJet 4i device (Eppendorf). For pneumococcus-VWF colocalization studies, RFP-bacteria were injected into the heart chamber of wild type larvae (WT Brassi PBS 0545, 5dpf). After 2 h of cultivation in Danioeau at RT, fixation of the larvae was performed by incubation in 4% PFA in PBS over night at 4°C. The immune fluorescence staining method of[11] was applied with minor modifications: Larvae were excised from the agarose and incubated in 200 µl PBS. After tissue perforation with acetone for 7 min at RT, larvae were washed for 5 min with ddH_2O, equilibrated for 5 min in PBS and incubated for 1 h with PBS-blocking buffer containing 2% goat serum (Roth), 3.0% BSA, and 1.0% DMSO. For detection of pneumococci, larvae were incubated over night at 4°C with a pneumococcus-specific rabbit antibody followed by incubation with a rabbit-specific Alexa 568-conjugated secondary antibody (Thermo Fisher Scientific). For VWF detection, a FITC-conjugated polyclonal anti-VWF-antibody from goat (Abcam) was used. Samples were washed with 3.0% BSA and 1.0% DMSO in PBS four times for 30 min in between each antibody incubation. For microscopic visualization, the stained larvae were embedded in 1.0% low melting agarose. For each study, a minimum of 12 larvae were injected in two independent experiments.

Legends for supplemental figures and videos

Figure S1. Detection of antibody background in dot spot analysis and peptide array analysis. Unspecific antibody binding to immobilized enolase **(A)** and enolase peptides **(B)** was detected by incubation of the membranes either with the hrp-conjugated mouse-specific secondary antibody alone (**B**, Control, secondary antibody) or with both, the primary VWF-specific mouse antibody and the hrp-conjugated secondary antibody (**A** and **B**, Control, first and secondary antibody). VWF overlay is shown for purposes of comparison. Only marginal unspecific antibody signals were detected. In between the two control experiments of the peptide array, residual antibodies were removed from the membrane by step wise incubation with a wash solution with urea followed by sonication in dimethylformamide.

Figure S2. Dot spot-antibody control and protein integrity of VWF domains A1, A2, A3. A) After immobilization of VWF A domains on nitrocellulose, the membrane was incubated with enolase specific antibodies and secondary antibodies in order to detect any unspecific background binding without incubation with enolase protein. **B)** Prior to binding studies, the purified VWF protein domains A1, A2, and A3 were subjected to SDS-page followed by Coomassie brilliant blue stain in order to confirm protein integrity. For each of the tested VWF domains, distinct protein bands were visualized (black arrows). The protein band representing domain A2 reveals a range of signals indicating that this protein is more sensitive to effects of degradation than domains A1 or A3. Therefore, only new protein preparations were used in binding analyses.

Figure S3. Zebrafish infection with RFP-expressing pneumococci. A) For visualization of pneumococcal circulation within the vascular system, RFP-expressing bacteria were injected into the heart chamber of stunned zebrafish larvae expressing red-fluorescing endothelium (FLK1:mCherryCAAX). Bacteria circulating in the vasculature and bacteria attached to the vessel walls were microscopically detected and are visualized in snap shots of three representative larvae (1, 2, and 3). The transparent tissue is shown in bright field (BF); endothelium and diplococcoid bacteria are visualized by red fluorescence at 540 nm/590 nm. The use of bacterial fluorescence dyes is restricted to RFP due to Zebrafish-tissue-derived autofluorescence. The overlay of fluorescence signals is shown in merged pictures. White arrows point to location of red fluorescent pneumococci. Videos of pneumococcal attachment

within larva no.3 are added in Supplements (Video 1-3). Scale bars represent 10 µm. **B)** Histogram of overlapping histograms from three different bacterial aggregates shown in Figure 4C (ROI 01, 02, 03, marked with red arrwos). The overlay of bright field and merged fluorescence signals (BF + merge) are shown in higher magnification for comparison with the histograms. Histograms visualize VWF-pneumococcus colocalization of three regions of interest (ROI 01, 02, 03). Scale bars represent 10 µm.

Figure S4. Representative mathematical fitting of binding kinetics. A) Binding of different concentrations of VWF full length protein to immobilized enolase and B) of VWF A1-domain to immobilized enolase. The 1:1 Langmuir model of the BIA evaluation software (Blck lines) was used to determine the dissociation constants shown in the supplementary Table S1.

Figure S5. Identification of a putative VWF binding site on the pneumococcal enolase. A) Localization of the putative VWF- binding pocket within the octameric enolase molecule depicts alternating enolase monomers colored in grey; the VWF-binding site is highlighted within one of the four dimers colored in grey. The whole VWF-binding peptide region no.3 representing aa 189 - 219 is marked in orange and the core peptide composed of aa 195 – 210 is marked in red. Front view is shown in A. B and C visualize the top view of the VWF-binding pocket in an enolase octamer. Structure visualization was calculated using PyMOL.

Figure S6. Microfluidic cell culture infection with RFP-expressing pneumococci in presence of heparin. After cultivation of HUVEC at a constant shear rate of 10 dyn/cm^2, heparin was added to the system followed by pneumococci. The supplementation of FITC-labelled VWF-specific antibodies enabled the detection and microscopic detection of VWF strings and aggregates on the cell surface. Even after 120 min of flow cultivation, no attachment of RFP-expressing pneumococci to the VWF strings could be detected. Fluorescence microscopy resulted in a representative illustration, which shows green fluorescing VWF strings (red arrow) at 470nm/515nm. No pneumococcal attachment could be detected using the filter 540nm/590nm, despite the constant monitoring of circulating fluorescent bacteria in the *live cell*-observation during the course of experimental analysis. The brightfield (BF) and the merged pictures of BF and green fluorescence indicated an unobtrusive cell morphology. The scale bar represents 10 µm.

Table S1. Kinetic parameters of Surface Plasmon Resonance evaluation. The 1:1 Langmuir model of the BIA evaluation software (Blck lines) was used to determine the dissociation constants of the interaction between VWF full length protein and immobilized enolase and between VWF A1-domain and immobilized enolase.

Table S2. Amino acid sequence comparison of the putative VWF binding site from different pathogenic bacteria and commensals. The table shows names of bacterial strains, the Accession numbers of the enolase proteins, the protein identity in percent and the peptide sequence of the putative VWF-binding motif. The identical amino acids are marked by a dot. The putative VWF binding peptide is conserved between *S. pneumoniae* D39 and R6 and shares 94 % sequence identity to the corresponding peptide of *S. aureus*.

Videos. Time lapse visualization of VWF recruitment and bacterial attachment within the blood flow of zebrafish larvae. Bacterial attachment to blood vessels is shown during pulsing blood circulation of the zebrafish mutant larva No. 3 (FLK1:mCherryCAAX). The corresponding region is depicted in Fig. 7A. **Video 1** monitors the blood circulation in the bright field channel, RFP-expressing diplococci attaching to the red fluorescence of the vascular endothelium is monitored in **Video 2**, and the merged channels are shown in **Video 3**. Each of the three videos represents 4 frames/min.
