# Supplementary material for: Von Willebrand Factor Mediates Pneumococcal Aggregation and Adhesion in Blood Flow
Source: Front Microbiol. 2019 Mar 26;10:511. doi: 10.3389/fmicb.2019.00511 (PMC6443961; doi:10.3389/fmicb.2019.00511)
Supplement: Supplementary file 7 [file Image_1.pdf]

Supplement

Fig. S1A

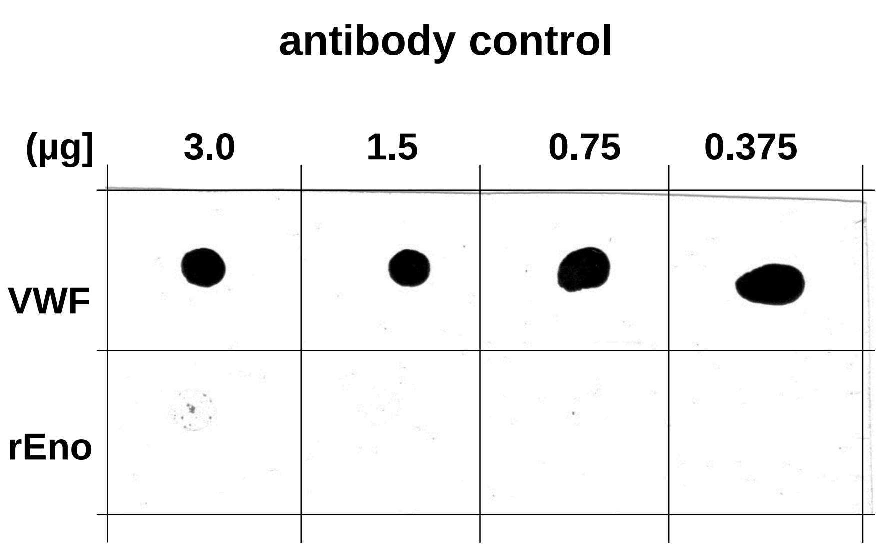

Fig. S1B

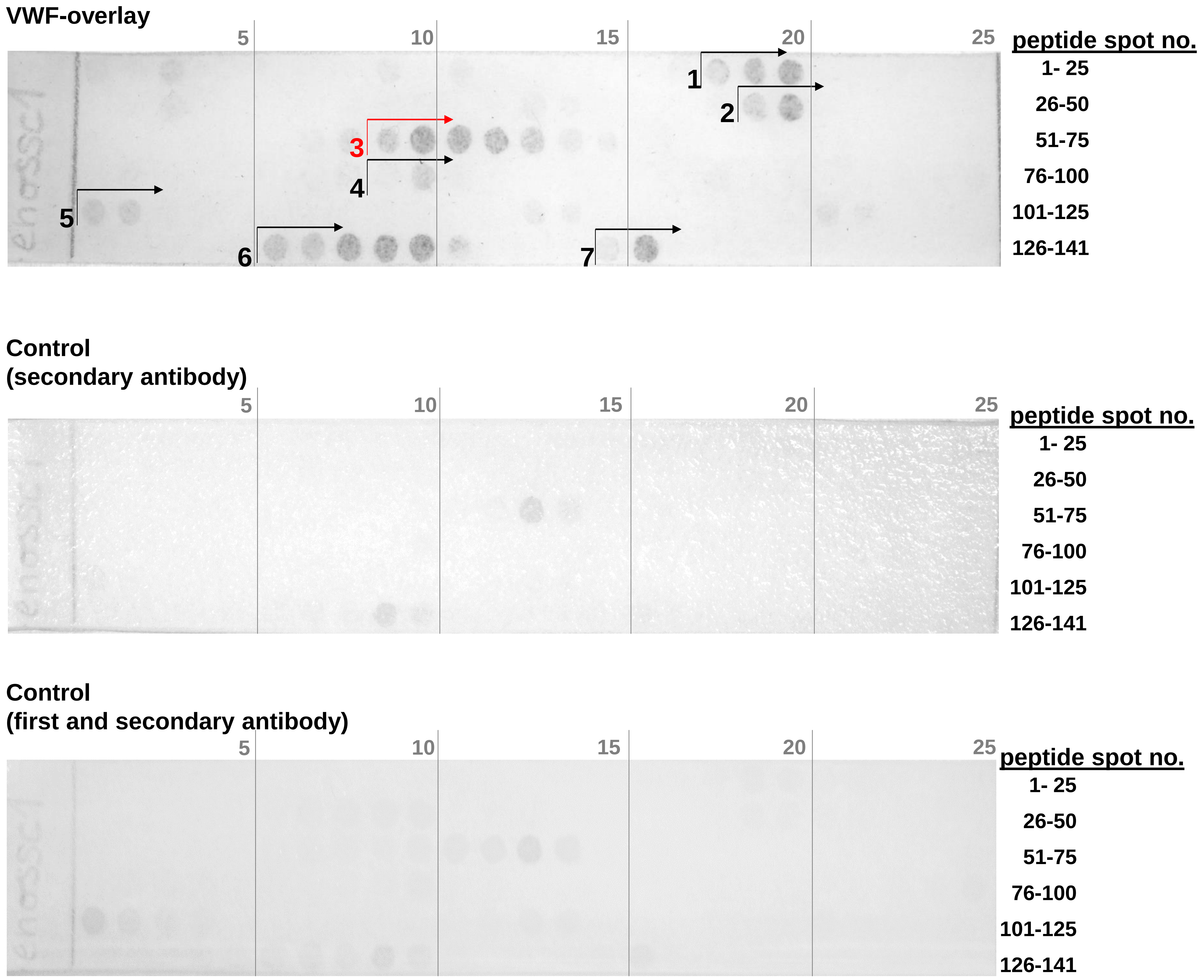

Figure S1. Detection of antibody background in dot spot analysis and peptide array analysis. Unspecific antibody binding to immobilized enolase **(A)** and enolase peptides **(B)** was detected by incubation of the membranes either with the hrp-conjugated mouse-specific secondary antibody alone (**B**, Control, secondary antibody) or with both, the primary VWF-specific mouse antibody and the hrp-conjugated secondary antibody (**A** and **B**, Control, first and secondary antibody). VWF overlay is shown for purposes of comparison. Only marginal unspecific antibody signals were detected. In between the two control experiments of the peptide array, residual antibodies were removed from the membrane by step wise incubation with a wash solution with urea followed by sonication in dimethylformamide.
