# Supplementary figures and images for: Von Willebrand Factor Mediates Pneumococcal Aggregation and Adhesion in Blood Flow
Source: Front Microbiol. 2019 Mar 26;10:511. doi: 10.3389/fmicb.2019.00511 (PMC6443961; doi:10.3389/fmicb.2019.00511)

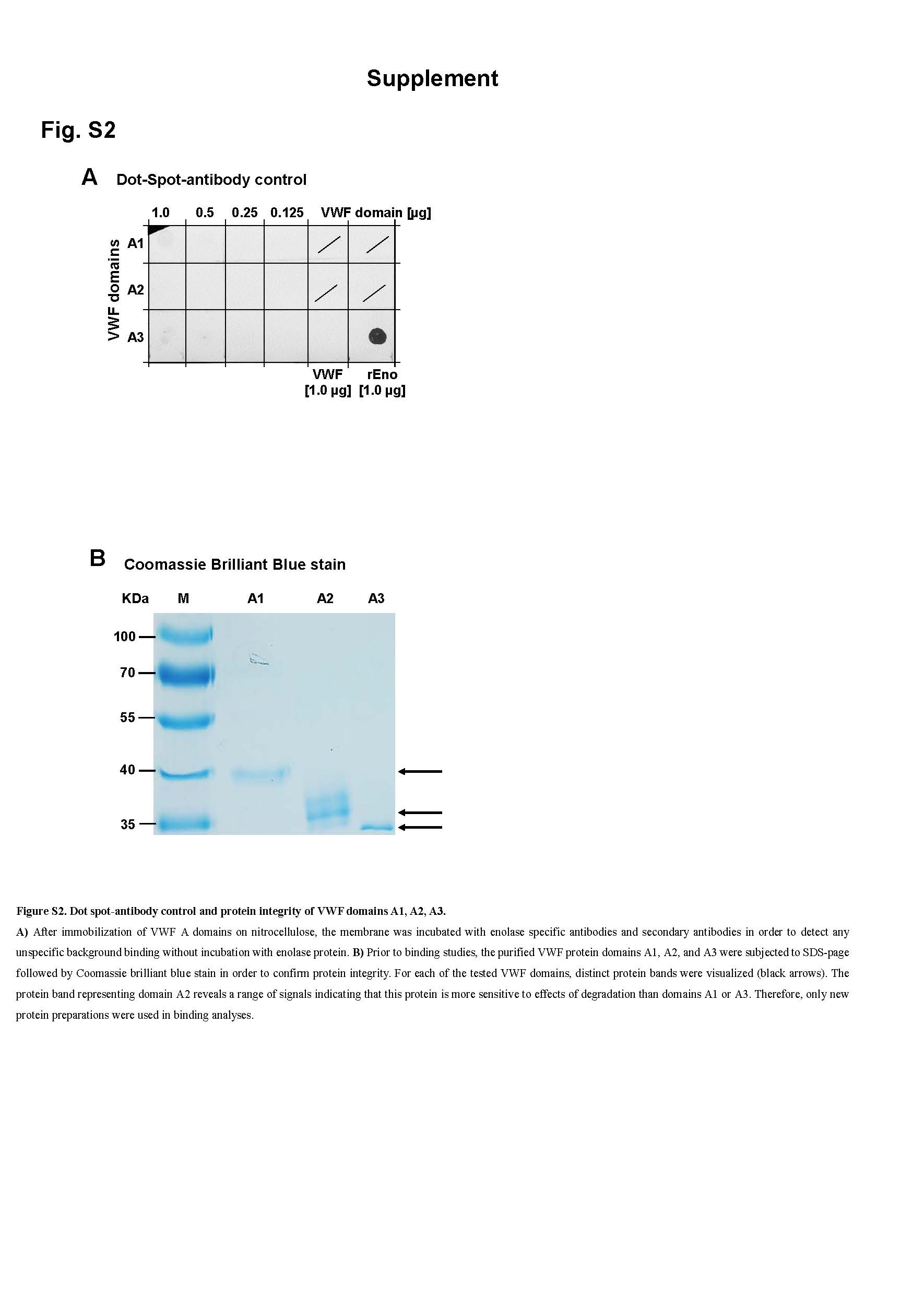

Supplement: Supplementary file 8 [file Image_2.JPEG]

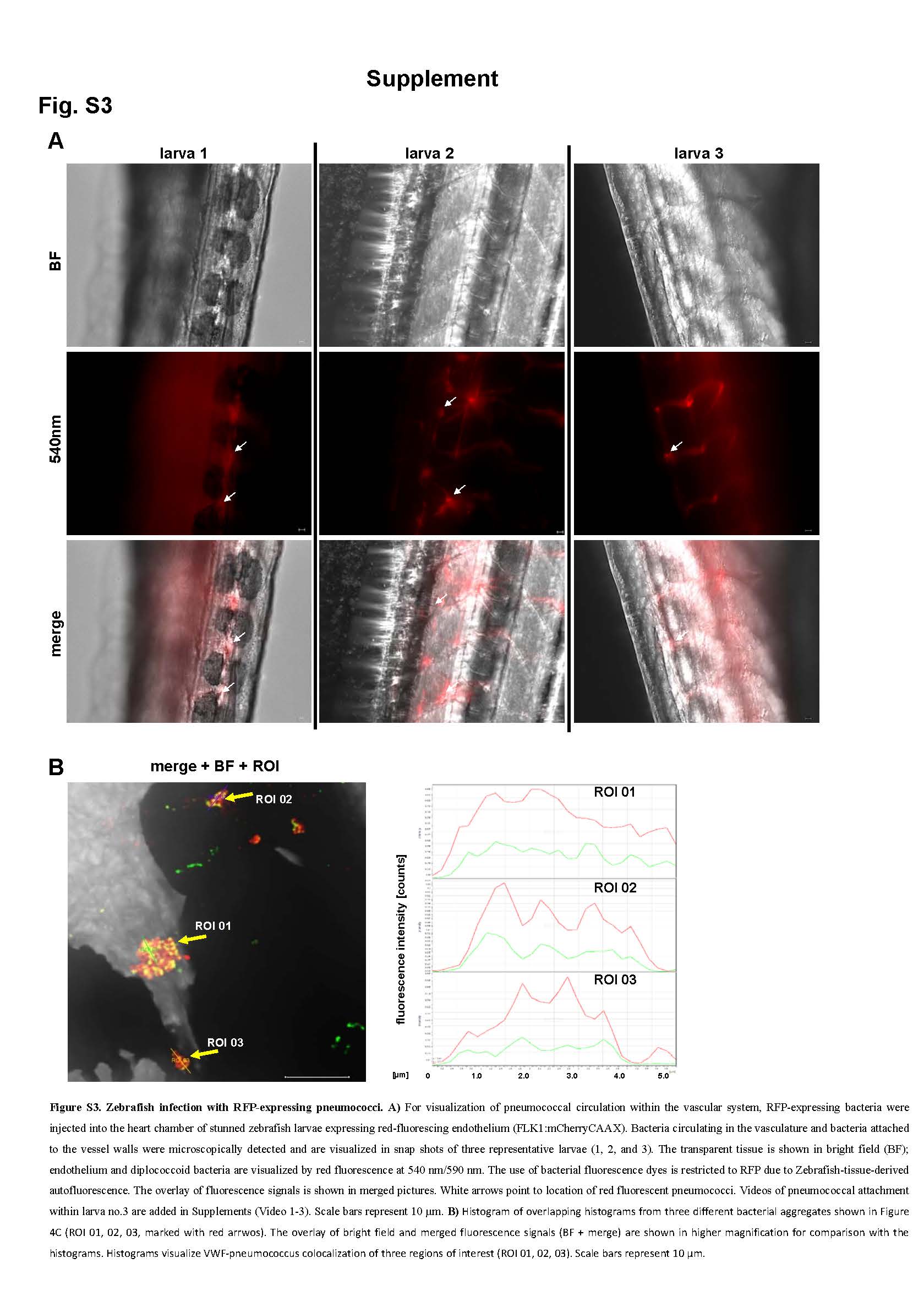

Supplement: Supplementary file 9 [file Image_3.JPEG]

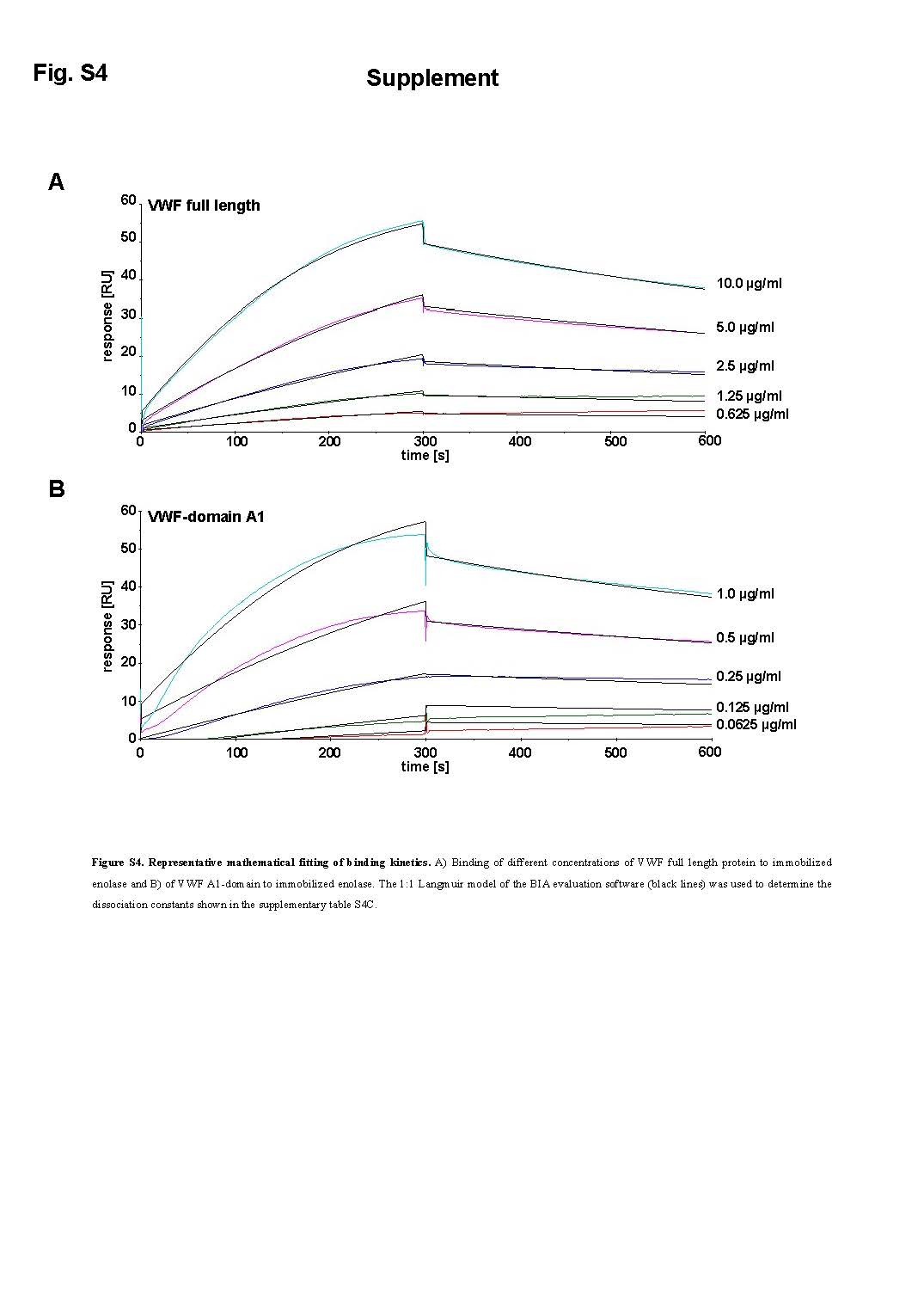

Supplement: Supplementary file 10 [file Image_4.JPEG]

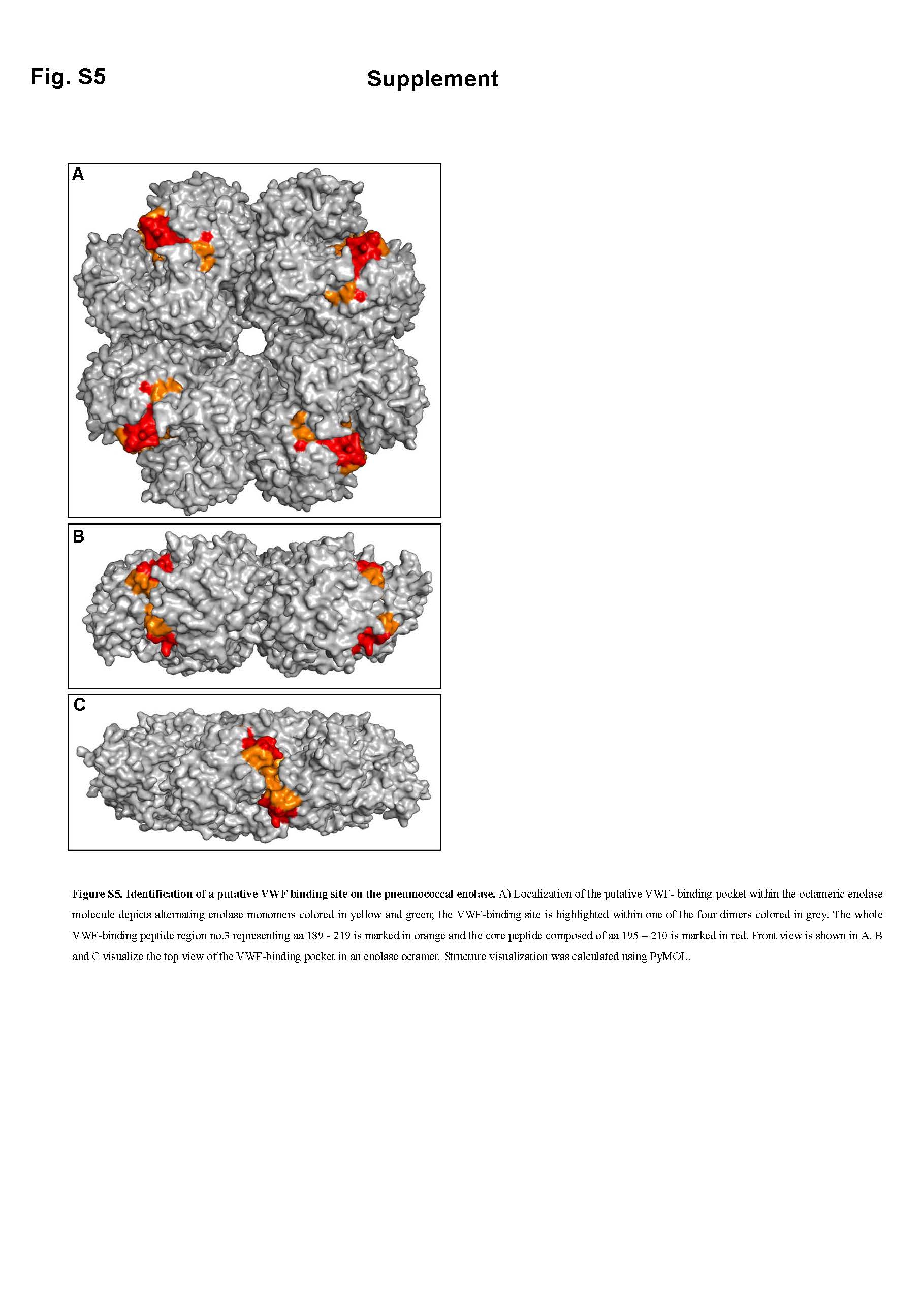

Supplement: Supplementary file 11 [file Image_5.JPEG]

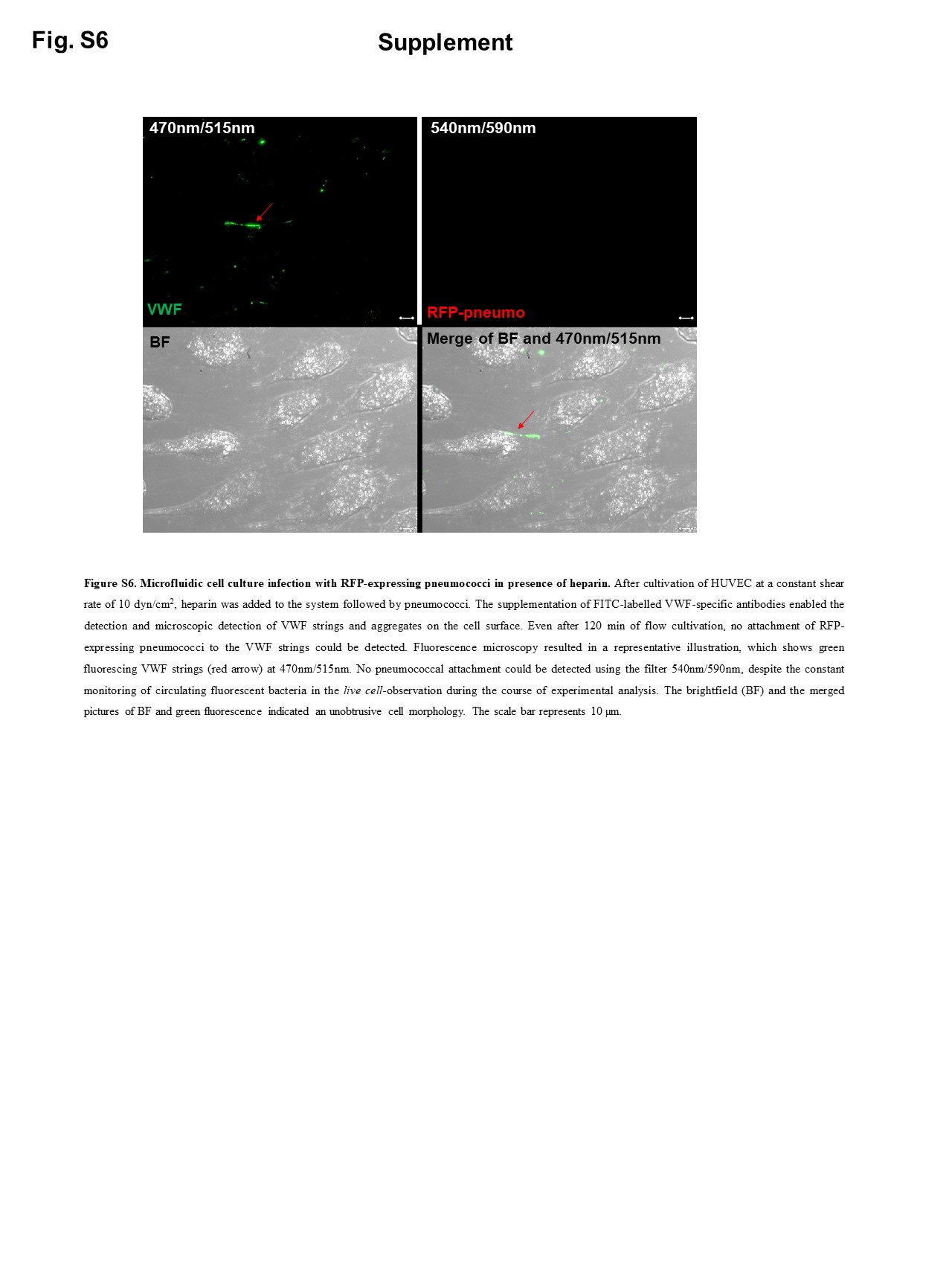

Supplement: Supplementary file 12 [file Image_6.jpg]
